# Natural killer cells and cytotoxic T lymphocytes are required to clear solid tumor in a patient-derived xenograft

**DOI:** 10.1172/jci.insight.140116

**Published:** 2021-07-08

**Authors:** Duy Tri Le, Tridu R. Huynh, Bryan Burt, George Van Buren, Shawn A. Abeynaike, Cristina Zalfa, Rana Nikzad, Farrah Kheradmand, John J. Tyner, Silke Paust

**Affiliations:** 1 Department of Pediatrics, Texas Children’s Hospital, Houston, Texas, USA.; 2 Scripps Research Translational Institute, La Jolla, California, USA.; 3 Division of Internal Medicine, Scripps Clinic/Scripps Green Hospital, La Jolla, California, USA.; 4 Department of Immunology and Microbiology, The Scripps Research Institute, La Jolla, California, USA.; 5 Division of General Thoracic Surgery and; 6 Division of Surgical Oncology, Michael E. DeBakey Department of Surgery, Baylor College of Medicine, Houston, Texas, USA.; 7 Dan L Duncan Comprehensive Cancer Center, Baylor College of Medicine, Houston, Texas, USA.; 8 Margaret M. and Albert B. Alkek Department of Medicine, Baylor College of Medicine and Michael E. DeBakey VA Medical Center, US Department of Veterans Affairs, Houston, Texas, USA.; 9 Division of Cardiovascular/Thoracic Surgery, Scripps Clinic, La Jolla, California, USA.

**Keywords:** Immunology, Cancer immunotherapy, NK cells, T cells

## Abstract

Existing patient-derived xenograft (PDX) mouse models of solid tumors lack a fully tumor donor–matched, syngeneic, and functional immune system. We developed a model that overcomes these limitations by engrafting lymphopenic recipient mice with a fresh, undisrupted piece of solid tumor, whereby tumor-infiltrating lymphocytes (TILs) persisted in the recipient mice for several weeks. Successful tumor engraftment was achieved in 83% to 89% of TIL-PDX mice, and these were seen to harbor exhausted immuno-effector as well as functional immunoregulatory cells persisting for at least 6 months postengraftment. Combined treatment with interleukin-15 stimulation and immune checkpoint inhibition resulted in complete or partial tumor response in this model. Further, depletion of cytotoxic T lymphocytes and/or natural killer cells before combined immunotherapy revealed that both cell types were required for maximal tumor regression. Our TIL-PDX model provides a valuable resource for powerful mechanistic and therapeutic studies in solid tumors.

## Introduction

There is mounting experimental evidence documenting the importance of effector functions mediated by cytotoxic T lymphocytes (CTLs) and natural killer (NK) cells in the host immune response to cancer, both individually and collaboratively (1–9). Activation of CTLs and NK cells has been linked to protective tumor immune surveillance, and higher numbers of tumor-infiltrating CTLs and/or NK cells are favorable prognostic indicators for many types of solid tumors (10), including lung cancer (11). T cell receptor ligation by tumor-derived antigens when presented on class I human leukocyte antigen (HLA), in combination with costimulatory receptors, activates and expands CTLs, which secrete cytokines and chemokines to lyse tumor cells (12). NK cells express a large number of activating and inhibitory receptors that mainly bind to self MHC-I (13, 14). This is essential in the context of cancer recognition because MHC-I is often downregulated by malignant cells, circumventing CTL-mediated killing (15). Furthermore, stress ligands expressed by tumor cells can activate NK cells through activating receptors, such as NKG2D (14, 16–18) and CD16, the FcγRIII responsible for antibody-dependent cellular cytotoxicity (19).

Immunosuppressive cells, such as myeloid-derived suppressor cells (MDSCs; ref. 20), M2 macrophages (21, 22), and regulatory T cells (Tregs; ref. 23), inhibit CTL effector and NK cell effector function in solid tumors (24) via the expression of inhibitory ligands, suppressive cytokines, and tumor-promoting factors (25). Indeed, the abundance of MDSCs and Tregs in solid tumors positively correlates with advanced disease and increased tumor burden (26–28) and is an independent predictor of poor outcome (29–32). Concordantly, pharmacological targeting of MDSCs and Tregs in animal models and human patients significantly improves antitumor immunity, enabling tumor control (33–36). In addition to the suppression of cytotoxic antitumor activities by immunosuppressive cells, tumors further trigger cell-intrinsic defects and the upregulation of immune checkpoint molecules on CTLs and NK cells via chronic overstimulation (37).

Immune checkpoint molecules have several nonredundant roles and distinct expression patterns required to prevent autoimmunity under normal conditions. In support of this concept, immune checkpoint–deficient mice display autoinflammatory diseases in multiple organs (38). A well-recognized mechanism of tumor evasion is the induction of immune checkpoint molecules, such as programmed death receptor-1 (PD-1) and its ligands (PD-L), that blunt T and NK cell effector function (39). Indeed, tumor-exhausted CTLs and NK cells express high levels of PD-1 (39), and tumor cells escape immune attack through the expression of PD ligands (40). Immune checkpoint inhibition (ICI) targeting these immunosuppressive pathways is FDA approved for the treatment of many solid tumors, including non-small cell lung cancer (NSCLC). However, blocking of immune checkpoint molecules alone does not guarantee tumor eradication by cytotoxic immune cells, as immune cell attack ultimately depends on the recognition of either tumor antigen by T cells or stress ligands by NK cells (41–43). This may explain why ICI is most effective in tumors with a high mutational burden, presumably resulting in an abundance of tumor antigens stimulatory to the immune system (44). Optimal elimination of tumor cells will, therefore, likely necessitate combining treatments with nonoverlapping mechanisms/targets. NK cell modulation is one such additive or alternative treatment modality. Many lines of evidence indicate that NK cells are crucial for tumor immunosurveillance/clearance. People deficient in NK cells suffer from recurrent viral infections and the development of various types of cancer (45). Additionally, the phenotype and number of NK cells in human tumors and animal models have been correlated with outcomes in various malignancies (46).

In mice and humans, IL-15 stimulation is required for NK cell development and memory CTL function (47, 48). Like IL-2, IL-15 is most effective when transpresented on the surface of antigen-presenting cells (macrophages, dendritic cells) attached to its α-receptor (IL-15Rα; ref. 49). Therefore, efforts aimed at optimizing IL-15 agonistic activity have culminated in the development of an IL-15 superagonist (ALT-803), which denotes the chimeric protein composed of IL-15 bound to the sushi domain of IL-15Rα and fused to the Fc domain of IgG1 (IL-15RαFc). Several studies have shown promising evidence in animal models of malignancy (50–52), and a phase I clinical trial demonstrated its safety (53). It is also noteworthy that IL-15 stimulation increases CTL and NK cell antitumor functions without expanding immunosuppressive Tregs (54).

Lung cancer is responsible for more than one-quarter of all cancer deaths worldwide (55). Lung adenocarcinoma (LUAD), a subtype of NSCLC, is a genetically heterogeneous cancer in part resulting from oncogenic mutations and the loss of tumor suppressors (56–60). The FDA has approved PD-1 or PD-L1 blockade for advanced NSCLC treatment (24, 31, 60, 61), although it is not a cure (62–65). Pancreatic ductal adenocarcinoma (PDAC) has a high proportion of immunosuppressive cells, such as Tregs and MDSCs (66), and its fibrous stroma expresses chemokines that trap CTLs (67–69). Due to its relatively low tumor mutational burden, PDAC is poorly immunogenic (44), has shown little susceptibility to ICI (70, 71), and is the fourth leading cause of cancer death in Western countries (72).

Existing patient-derived xenograft (PDX) models lack a human tumor donor–matched, competent, and mouse-tolerant immune system (73–78). This key limitation renders these systems nonoptimal regarding immunomodulatory studies of a human tumor’s endogenous immune components. To fulfill this unmet need, we developed a PDX model of human solid tumor with a syngeneic, competent, mouse-tolerant, and functional tumor-infiltrating lymphocyte–derived (TIL-derived) immune system that reconstitutes recipient mice for at least 6 months after tumor engraftment. We show that these immune cells are responsive to combination immunotherapy and allow for in-depth mechanistic in vivo studies. Specifically, using our TIL-PDX model of human lung adenocarcinoma (TIL-PDX-LUAD), we show profound tumor regression when treated with a combination immunotherapy consisting of IL-15 stimulation and PD-1 blockade. We further identified NK cells and CTLs to both be required for optimal tumor regression using this combination immunotherapy. Our TIL-PDX model represents a valuable resource for powerful preclinical mechanistic and therapeutic studies of solid tumors.

## Results

### NK cells in human LUAD express PD-1 and PD ligands.

**Figure 1:**
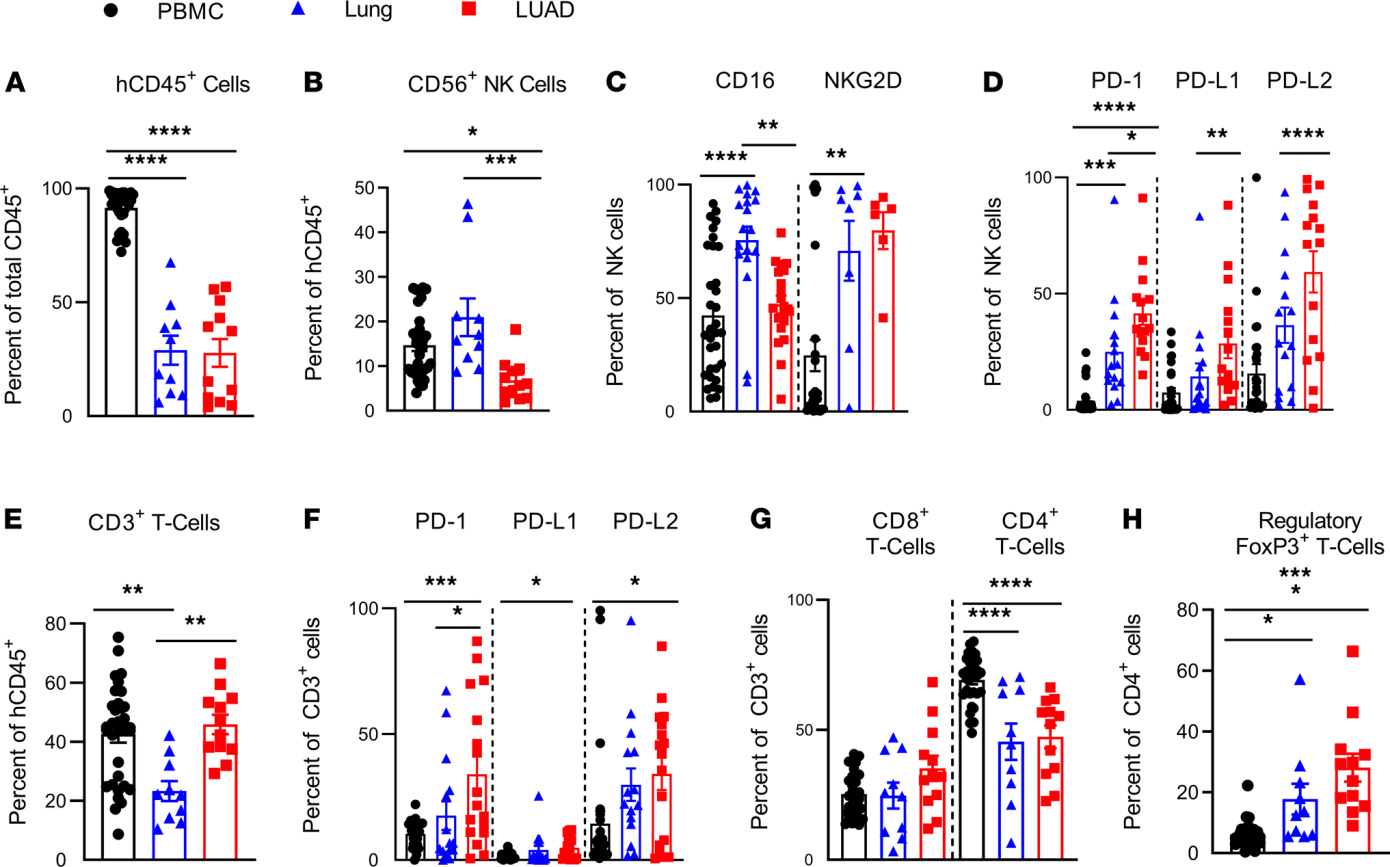
Human LUAD-associated NK cells and CD3^+^ T cells are exhausted in phenotype. Immunophenotyping of human NK cells and T cells in 3 different human tissues at the time of tumor excision: human donor LUAD, tumor donor–matched normal lung, and tumor donor-matched PBMCs. (**A**) Frequency of total human leukocytes (human CD45^+^); (**B**) frequency of human NK cells (hCD45^+^CD3^–^CD56^+^); (**C**) frequency of CD16- and/or NKG2D-expressing human NK cells; (**D**) frequency of PD-1–, PD-L1–, and/or PD-L2–expressing human NK cells; (**E**) frequency of CD3^+^ T cells; (**F**) frequency of PD-1–, PD-L1–, and/or PD-L2–expressing human CD3^+^ T cells; (**G**) frequency of CD8^+^ T cells (hCD45^+^CD3^+^CD8^+^) and CD4^+^ T cells (hCD45^+^CD3^+^ CD4^+^); and (**H**) frequency of Tregs (hCD45^+^CD3^+^CD4^+^Foxp3^+^) as percentage of the CD4^+^ T cell subset. Each data point represents 1 genetically unrelated human donor; *n* = 6 to 33. Mean ± SEM shown. One-way ANOVA with post hoc Tukey’s test. *P < 0.05; **P < 0.01; ***P < 0.001, ****P < 0.0001.

We first used flow cytometry to evaluate the phenotypes of NK cells in various tissues of the human donors, which consisted of (a) the lung tumor (LUAD), (b) donor-matched normal lung tissue (lung), and (c) donor-matched PBMCs (PBMCs). The frequency of total CD45^+^ leukocytes was significantly decreased in LUAD and donor-matched normal lung tissue compared with PBMCs. There was, however, no difference in total CD45^+^ leukocytes between LUAD and donor-matched normal lung tissue (Figure 1A). Of these leukocytes, the frequency of NK cells was significantly decreased in LUAD, and fewer of these LUAD-resident NK cells expressed the activating receptor CD16 compared with donor-matched normal lung tissue (Figure 1, B and C). Notably, there was a significantly higher proportion of tumor-resident NK cells expressing PD-1 and/or its ligands, PD-L1 and PD-L2, when compared with normal lung– or PBMC-derived NK cells (Figure 1D). We unexpectedly found high numbers of PD-L1– and PD-L2–expressing NK cells in both nontumor lung tissue and LUAD, and this was consistent across genetically unrelated human donors (Supplemental Figure 1H; supplemental material available online with this article; https://doi.org/10.1172/jci.insight.140116DS1). These data suggest that PD-1, PD-L1, and PD-L2 are expressed on lung tissue, and LUAD-resident NK cells and a significantly higher number of NK cells express PD-1, PD-L1, and/or PD-L2 in LUAD compared with nontumor lung tissue.

### T cells in human LUAD express PD-1 and PD-L2.

In contrast to NK cells, we found that T cells were more frequent in tumor tissue than nontumor lung tissue of the same donor when analyzed on the day of surgery (Figure 1E). As expected, PD-1 expression was increased in lung tumor-infiltrating T cells compared with donor-matched lung tissue, and T cells in both normal lung tissue and tumors expressed significantly higher levels of PD-L2 (Figure 1F). PBMC-derived T cells did not express significant levels of PD family members, including either PD ligand (Figure 1F). These data suggest that T cells, similar to NK cells, express PD-1 and/or PD ligands when residing in lung tissue and/or lung tumor, but not in peripheral blood in humans. These findings were consistent across genetically unrelated human donors (Supplemental Figure 1C).

### Generation of PDX of human LUAD.

**Figure 2:**
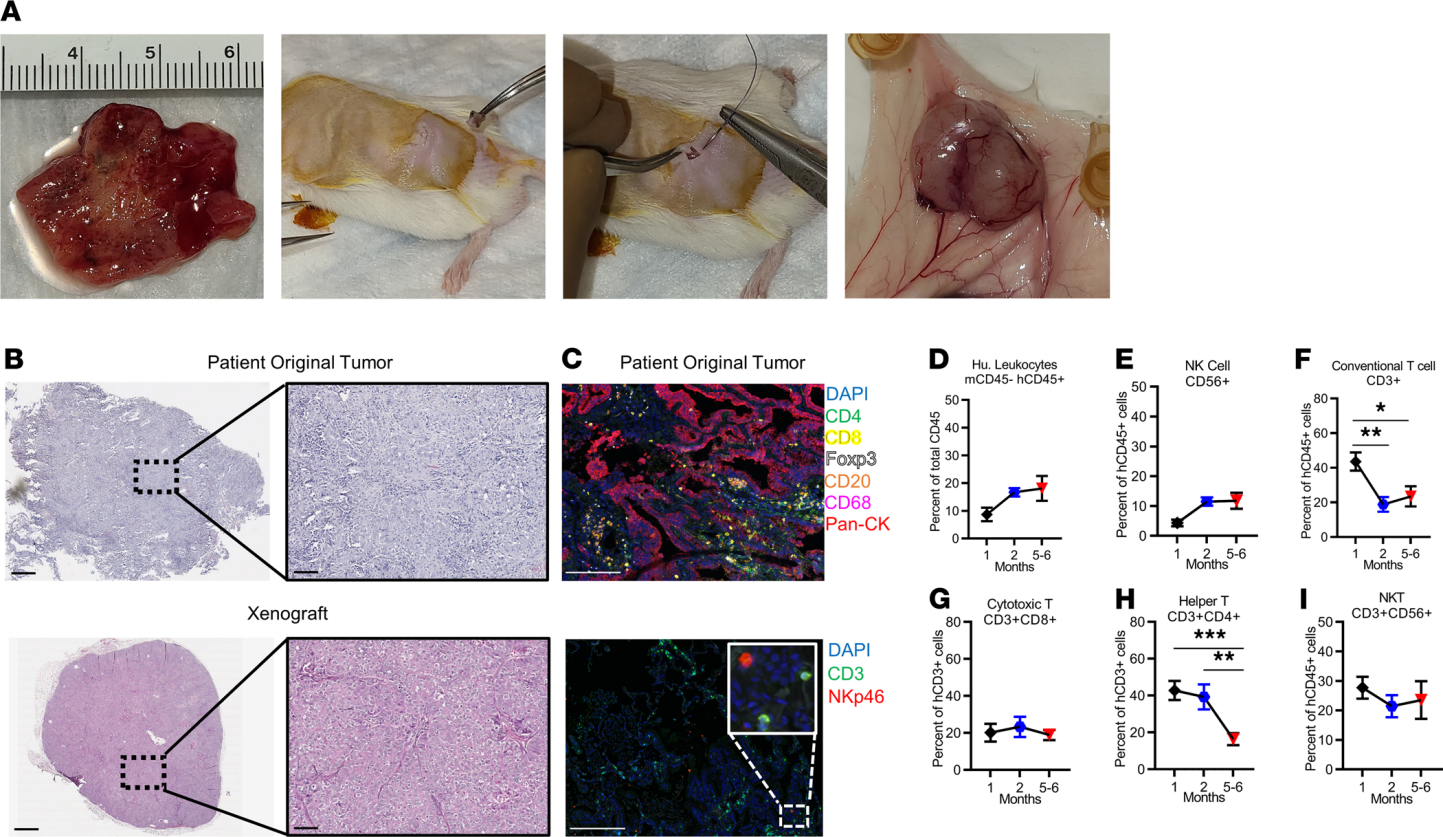
Human immune cells persist for months in TIL-PDX-LUAD mice. (**A**) Generation of the TIL-PDX model of human LUAD in NSG mice: human lung adenocarcinoma freshly dissected from lobectomy, single 2 to 3 mm^3^ piece of undisrupted tumor inserted into the subcutaneous space of an NSG mouse flank, simple interrupted sutures or surgical clips used to close the incision site, and vascularized tumor as early as 6 weeks postimplantation. (**B**) Representative H&E staining at 10× and associated 40× magnifications of human donor LUAD and TIL-PDX–excised LUAD demonstrating preserved carcinoma architecture. (**C**) Top: 7-color IHC stain (DAPI, CD4, CD8, Foxp3, CD20, CD68, pan-cytokeratin) of a patient’s original tumor prior to implant. Bottom: CD3, NKp46, DAPI IHC stain of a patient’s original tumor prior to implant. (**D**–**I**) Flow cytometry of human immune cells from PBMC of TIL-PDX-LUAD mice, 1 month, 2 months, or 5–6 months posttransplant. Percentage of (**D**) human hematopoietic cells (human CD45^+^) of total (mouse CD45^+^ + human CD45^+^) hematopoietic cells; (**E**) NK cells (CD45^+^CD3^–^CD56^+^); (**F**) conventional T cells (CD45^+^CD3^+^CD56^–^), including (**G**) CTLs (CD45^+^CD3^+^CD4^–^CD8^+^CD56^–^) and (**H**) T helper (CD45^+^CD3^+^CD4^+^CD8^–^CD56^–^) subsets; and (**I**) NK T cells at indicated times posttransplant. Scale bars: 800 μm for main panels, 100 μm for insets (**B**); 200 μm (**C**). **D**–**I**: Six genetically unrelated donor cohorts were analyzed 1 and 2 months after transplantation. Depending on cohort size, 3 to 7 TIL-PDX-LUAD mice were analyzed from each donor cohort at each time point resulting in *n* = 15 to 42 data points per cell type and time point. As our IACUC requires euthanasia of PDX mice whose tumors reach a specified tumor size, we analyzed 4 unrelated donor cohorts 6 months after transplantation. Depending on cohort size, 5 to 9 TIL-PDX-LUAD mice were analyzed per cohort, resulting in 23 to 39 data points per cell type. Data are represented as mean ± SEM; 1-way ANOVA with post hoc Tukey’s test. *P < 0.05; **P < 0.01; ***P < 0.001.

To generate our TIL-PDX-LUAD model, we transplanted a single, undisrupted piece of freshly resected LUAD from treatment-naive human donors into the subcutaneous space of a recipient NOD/SCID/IL2Rγ_c_-KO (NSG) mouse (Figure 2A). About 6 weeks later, the lung tumors had engrafted in about 70% of NSG mice with extensive vascularization when human donors and recipient NSG mice were sex matched. Importantly, histological comparison of input human LUAD versus the tumor status after implantation in the NSG mice for more than 3 months demonstrated preservation of original carcinoma architecture and nuclear atypia (Figure 2B). Using flow cytometry, we examined the breadth and longevity of TIL-derived reconstitution in the NSG mouse recipient’s peripheral blood (PB). We examined the frequencies of total human CD45^+^ cells, NK cells, and conventional T cells (including T helper, CTL, and Treg), 1, 2, and 6 months after tumor implantation. We found that all of these immune cell types were present in TIL-PDX-LUAD mice for at least 6 months (Figure 2, D–I). As in humans, NK cells made up 5% to 10% of PB hematopoietic cells in TIL-PDX-LUAD mice, with a predominance of human T lymphocytes. T cells were composed of CD4^+^ T helper and CD8^+^ CTLs at a 2:1 ratio. Notably, the ratio of NK/T cells and DCs/T cells did not differ between the fresh LUAD input tissues and the excised LUADs’ status after implantation in NSG mice (Supplemental Figure 2). Detailed analysis of our largest cohort revealed results consistent as previously stated and a relatively preserved ratio from mouse to mouse in terms of NK to CD3^+^ T cells (Supplemental Figure 3B). Additionally, B cells and DCs were also present in TIL-PDX-LUAD mice (Supplemental Figure 4). Further strengthening our analysis, multiplex immunohistochemical (IHC) staining of the donor lung tumor revealed the landscape of immune cells within the tumor stroma, not in peri-lymphoid structures. Most immune cell subsets were seen to be represented in the tumor stroma, including CD4^+^ and CD8^+^ T cells, Tregs, B cells, macrophages, and NK cells (Figure 2C).

We did not transfer any cells into the mice, whether it be patient-derived PBMCs or stem cells. All the immune cells perceived were derived from the TILs accompanying the implanted piece of tumor. Notably, most TIL-PDX-LUAD from a single donor developed robust reconstitution with a competent TIL-derived immune system in a cohort of 22 mice (Supplemental Figure 3A). Altogether, these data demonstrate long-term reconstitution with a TIL-derived, syngeneic, human immune system and underscore the successful development of a PDX model in which immune cells are donor matched to the tumor. Because tumors generally become palpable and start growing noticeably around 4 to 6 weeks posttransplant, this immune cell reconstitution timeline allows for several months of immunotherapy studies in our TIL-PDX-LUAD model.

### Human NK and T cells in TIL-PDX-LUAD mice resemble TILs of LUAD donors.

**Figure 3:**
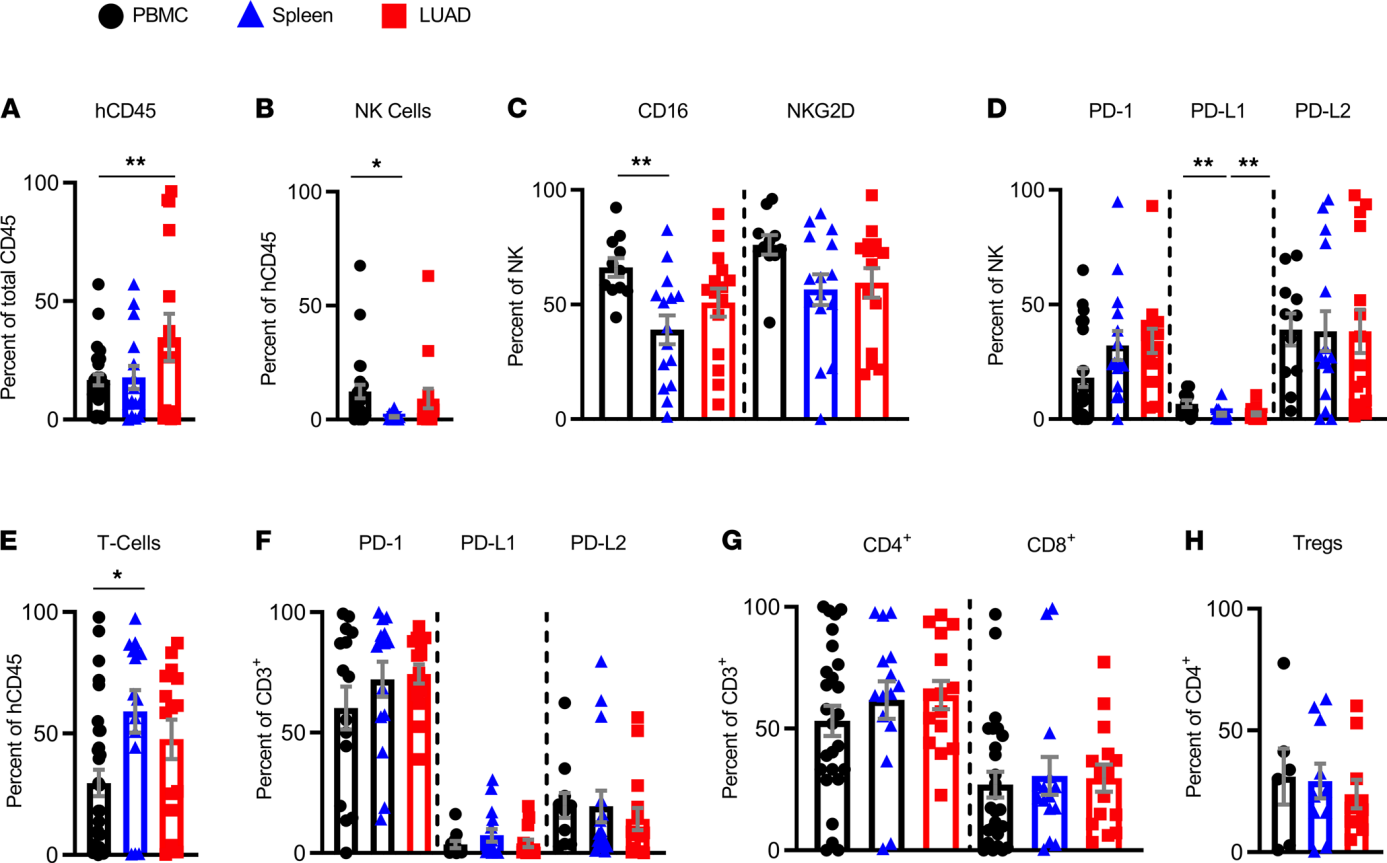
Reconstituted TILs in TIL-PDX-LUAD mice retain human donor LUAD immune phenotypes. Flow cytometry of human immune cells from PBMCs, spleen, and transplanted LUAD of TIL-PDX mice analyzed 2 to 3 months posttransplant. (**A**) Frequency of human hematopoietic cells and (**B**) human NK cells (CD45^+^CD56^+^CD3^–^) and (**C**) expression of activating receptors CD16 and NKG2D, as well as (**D**) PD-1, PD-L1, and PD-L2 on indicated tissues. (**E**) Frequency of conventional T cells (CD45^+^CD56^–^CD3^+^) and (**F**) their expression of PD-1, PD-L1, and PD-L2. (**G**) Frequencies of T helpers (CD45^+^CD3^+^CD4^+^CD8^–^) and CTLs (CD45^+^CD3^+^CD4^–^CD8^+^) as percentage of CD3^+^ cells and (**H**) Tregs (CD45^+^CD3^+^CD4^–^CD8^+^Foxp3^+^) as percentage of the CD4^+^ T cell subset in indicated tissues of TIL-PDX-LUAD mice. Five genetically unrelated donor cohorts were analyzed. Depending on cohort size, 3 to 6 TIL-PDX-LUAD mice of each cohort were analyzed at each time point, resulting in *n* = 15 to 30 data points total per cell type/marker and time point. Data are represented as mean ± SEM; 1-way ANOVA with post hoc Tukey’s test; *P < 0.05; **P < 0.01.

We analyzed NK cells and T cells’ proportion and immunophenotype in 3 organs of the TIL-PDX-LUAD (PBMC, spleen, and LUAD) 2 to 3 months postimplant. Human hematopoietic cells (human CD45^+^) were seen to be present in all 3 compartments (Figure 3A), including NK and CD3^+^ T cells (Figure 3 A, B, and E). Notably, NK cells retained expression of CD16 and NKG2D (Figure 3C), as well as PD receptors and ligand, namely PD-1 and PD-L2, similar to the input human LUAD from which they were derived (Figure 3D). As for subsets of CD3^+^ T cells, CD4, CD8, and Tregs continued to be present, and these cells continued to express PD-1 (Figure 3, F–H).

We also compared human donor PBMCs and LUAD on the day of excision versus TIL-PDX-PBMC and TIL-PDX-LUAD 2 to 3 months postimplant. One caveat to this analysis is that the TIL-PDX–derived PBMCs and LUAD were analyzed 2 to 3 months postimplantation into NSG mice. Therefore, they may not fully reflect what the tumor or PBMCs would have looked like had they remained in the patient and been analyzed from the patient 2 to 3 months later. Through this analysis, we found that there was a decrease in total leukocytes between input human LUAD and PBMCs when compared with TIL-PDX-LUAD and PBMCs after implantation (Supplemental Figure 5A). The proportion of CD16-, NKG2D-, and PD-1–expressing NK cells or T cells was largely preserved between the input human LUAD and TIL-PDX-LUAD 2 to 3 months postimplant (Supplemental Figure 5).

As far as nonhematopoietic cells in the tumor (CD45^–^), we observed reduced PD-L1 expression in TIL-PDX-LUAD compared with freshly excised LUAD, while PD-L2 expression was preserved (Supplemental Figure 6, A and B). As our freshly excised and TIL-PDX–derived tumor tissues were not always from the same donor, it is not possible to determine whether this reduction in PD-L1 and increase in PD-1 expression are due to patient-to-patient variation or whether factors that drive PD-L1/PD-1 expression on human NK cells, T cells, and LUAD in humans are reduced in TIL-PDX mice. We conclude that LUAD-resident NK and T cells survive long-term in TIL-PDX mice, in which they repopulate peripheral tissues and essentially maintain their tumor-associated phenotypes.

### Establishment of the TIL-PDX model with PDAC.

**Figure 4:**
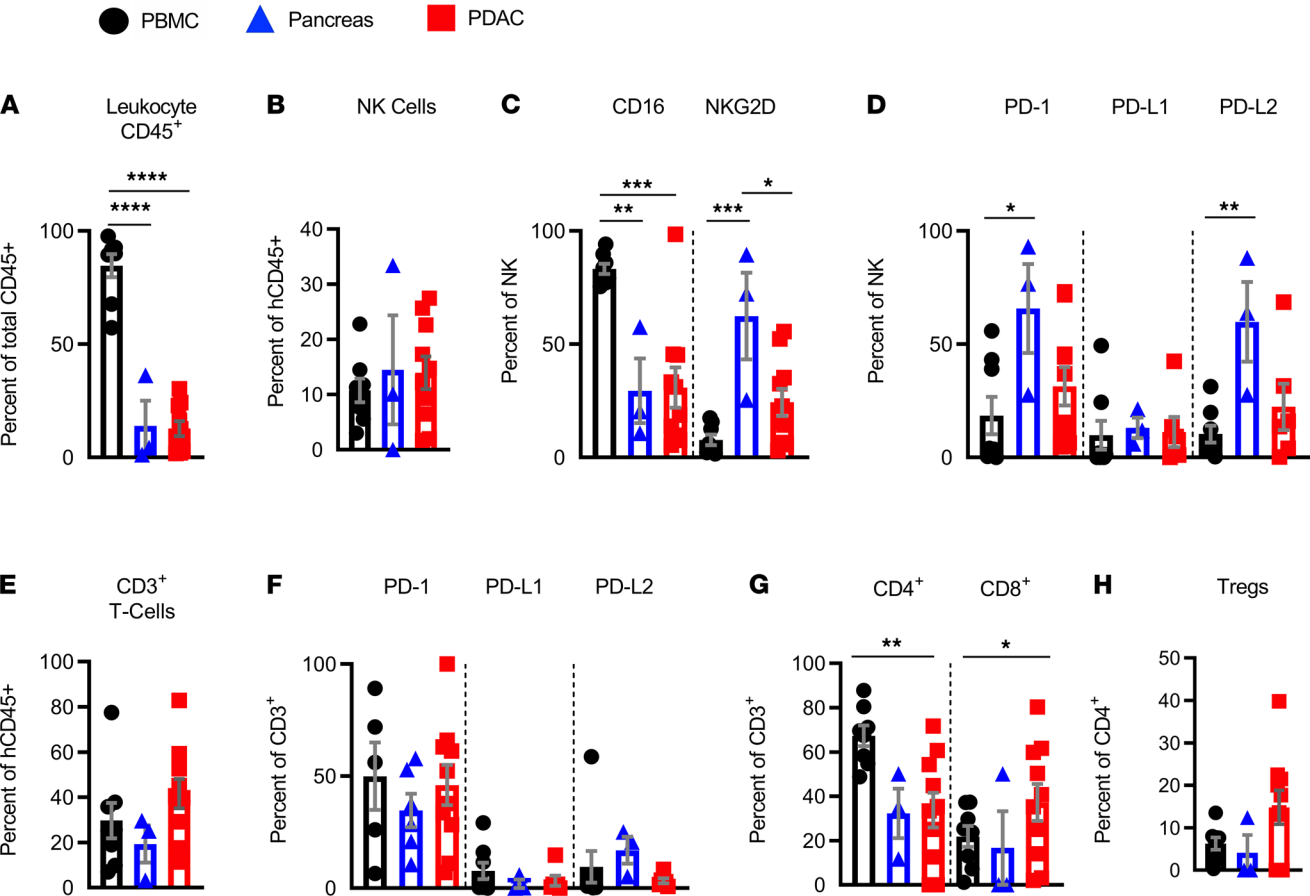
Human donor PDAC–associated NK and CD3^+^ T cells express PD-1 and NK cells as well as PD-L2. Immunophenotyping of human NK cells and T cells in 3 different human tissues at the time of tumor excision: human donor PDAC, tumor donor–matched normal pancreas, and tumor donor–matched PBMCs. (**A**) Frequency of total human leukocytes (human CD45^+^); (**B**) frequency of human NK cells (hCD45^+^CD3^–^CD56^+^); (**C**) frequency of CD16- and/or NKG2D-expressing human NK cells; (**D**) frequency of PD-1–, PD-L1–, and/or PD-L2–expressing human NK cells; (**E**) frequency of CD3^+^ T cells; (**F**) frequency of PD-1–, PD-L1–, and/or PD-L2–expressing human CD3^+^ T cells; (**G**) frequency of CD8^+^ T cells (hCD45^+^CD3^+^CD8^+^) and CD4^+^ T cells (hCD45^+^CD3^+^CD4^+^); and (**H**) frequency of Tregs (hCD45^+^CD3^+^CD4^+^Foxp3^+^) as percentage of the CD4^+^ T cell subset. Each data point represents 1 genetically unrelated human donor; *n* = 3 to 12. Mean ± SEM shown. One-way ANOVA with post hoc Tukey’s test. *P < 0.05; **P < 0.01; ***P < 0.001, ****P < 0.0001.

To support the future development of improved immunotherapies for human PDAC, we evaluated whether it was suitable for the generation of TIL-PDX mice. To do so, we engrafted NSG mice with a single piece of undisrupted, treatment-naive PDAC tissue and subsequently evaluated tumor growth, immune cell reconstitution, and immune phenotypes of T and NK cells by flow cytometry. When comparing input human PDAC, donor-matched normal pancreas, and donor-matched PBMCs, we observed fewer total leukocytes in PDAC or donor-matched normal pancreas when compared with PBMCs. However, their level was similar when compared with each other (Figure 4A). Proportions of NK cells were relatively equal across all 3 tissues (Figure 4B). Looking at NK cell-activating receptors, we found significantly fewer CD16-expressing cells in PDAC or normal pancreas than PBMCs, while their levels were comparable between PDAC and normal pancreas. Furthermore, while NKG2D-expressing NK cells were abundant in the normal pancreas, they were sparser in PBMCs and PDAC (Figure 4C). Similar to PD-1 and PD-L2 expression on lung tissue–resident NK cells, pancreatic tissue–resident NK cells expressed PD-1 and PD-L2. However, in contrast to lung tissue, PD-L1 expression was not different between PBMCs, pancreas, or PDAC-resident NK cells (Figure 4D). Interestingly, T cells expressed PD-1 but not the PD ligands in all 3 tissues (Figure 4F). Like LUAD, total T cells (Figure 4E), T helper cells, and CTLs (Figure 4G) were increased in frequency in the tumor compared with nontumor pancreatic tissue, presumably due to increased immunosuppressive Tregs (Figure 4H).

**Figure 5:**
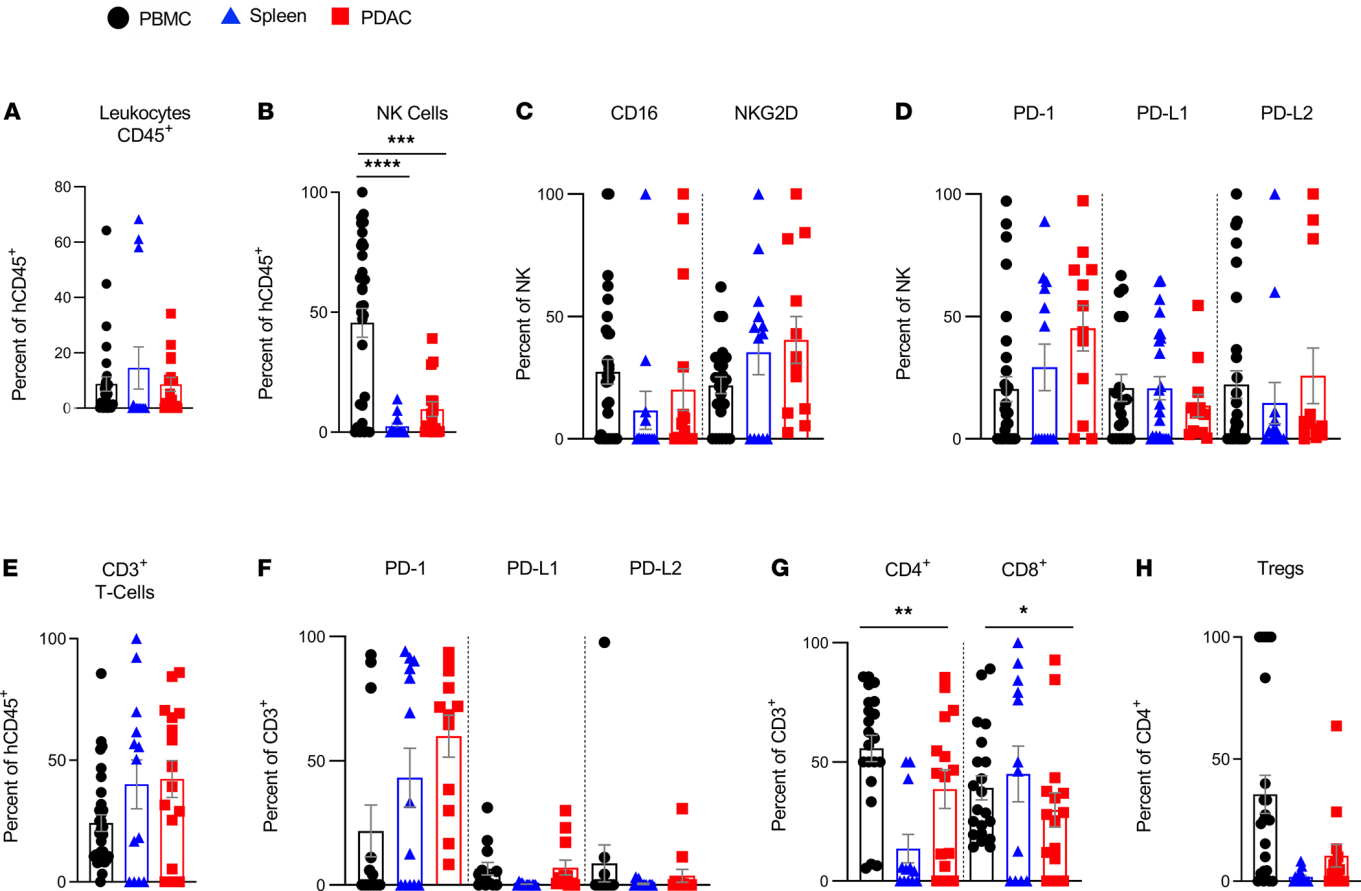
Reconstituted TILs in TIL-PDX-PDAC mice retain human donor PDAC immune phenotypes. Flow cytometry of human immune cells from PBMCs, spleen, and transplanted PDAC of TIL-PDX-PDAC mice analyzed 4 to 6 months posttransplant. (**A**) Frequency of total human leukocytes (human CD45^+^); (**B**) frequency of human NK cells (hCD45^+^CD3^–^CD56^+^); (**C**) frequency of CD16- and/or NKG2D-expressing human NK cells; (**D**) frequency of PD-1–, PD-L1–, and/or PD-L2–expressing human NK cells; (**E**) frequency of CD3^+^ T cells; (**F**) frequency of PD-1–, PD-L1–, and/or PD-L2–expressing human CD3^+^ T cells; (**G**) frequency of CD8^+^ T cells (hCD45^+^CD3^+^CD8^+^) and CD4^+^ T cells (hCD45^+^CD3^+^CD4^+^); and (**H**) frequency of Tregs (hCD45^+^CD3^+^CD4^+^Foxp3^+^), as percentage of the CD4^+^ T cell subset in indicated tissues of TIL-PDX-PDAC mice. PBMC: Five genetically unrelated donor-cohorts were analyzed. Depending on cohort size, 3 to 6 TIL-PDX-PDAC mice of each cohort were analyzed, resulting in 15 to 30 data points total per cell type/marker and time point. Spleen and transplanted PDAC: Four genetically unrelated donor cohorts were analyzed. Depending on cohort size, 3 to 5 TIL-PDX-PDAC mice of each cohort were analyzed at each time point, resulting in 12–20 data points total per cell type/marker and time point. Data are represented as mean ± SEM; 1-way ANOVA with post hoc Tukey’s test. *P < 0.05; **P < 0.01; ***P < 0.001; ****P < 0.0001.

Similar to TIL-PDX-*LUAD* mice, analysis of TIL-PDX-*PDAC* mice 2–4 months after implantation revealed the persistence of human hematopoietic cells (Figure 5A), which included effector cells such as NK cells (Figure 5, B–D) and conventional T cells (Figure 5, E and F). Subclassification of T cells revealed the presence of T helpers, CTLs, and Tregs (Figure 5, G and H). Tregs, MDSCs, and M2 macrophages that expressed TGF-β and/or IL-10 were also present in TIL-PDX-PDAC and TIL-PDX-LUAD’s PB and peripheral tissues 4 to 6 months after tumor implantation (Supplemental Figure 7, A–L). TIL-PDX-PDAC–resident NK cells expressed varying levels of CD16, NKG2D, PD-1, PD-L1, and PD-L2, and they retained their exhausted phenotype (Figure 5, C and D). Similarly, T cells, including substantial amounts of Tregs, persisted in TIL-PDX-PDAC mice and expressed PD-1, but not PD-L1 or PD-L2, resembling the PDAC donor tissues from which they were derived (Figure 5, E and F). Also preserved were PD-L1 and PD-L2 expression on PDAC tumor cells, as freshly excised PDAC expressed PD-L1 and PD-L2, and expression was maintained after implantation in xenografts (Supplemental Figure 6, C and D). We conclude that TIL-PDX mice generated with LUAD or PDAC reconstitute an immune system resembling that of the human donor’s TILs. This immune reconstitution lasts for several months.

### Correlation between tumor growth and immune cells in TIL-PDX model.

**Figure 6:**
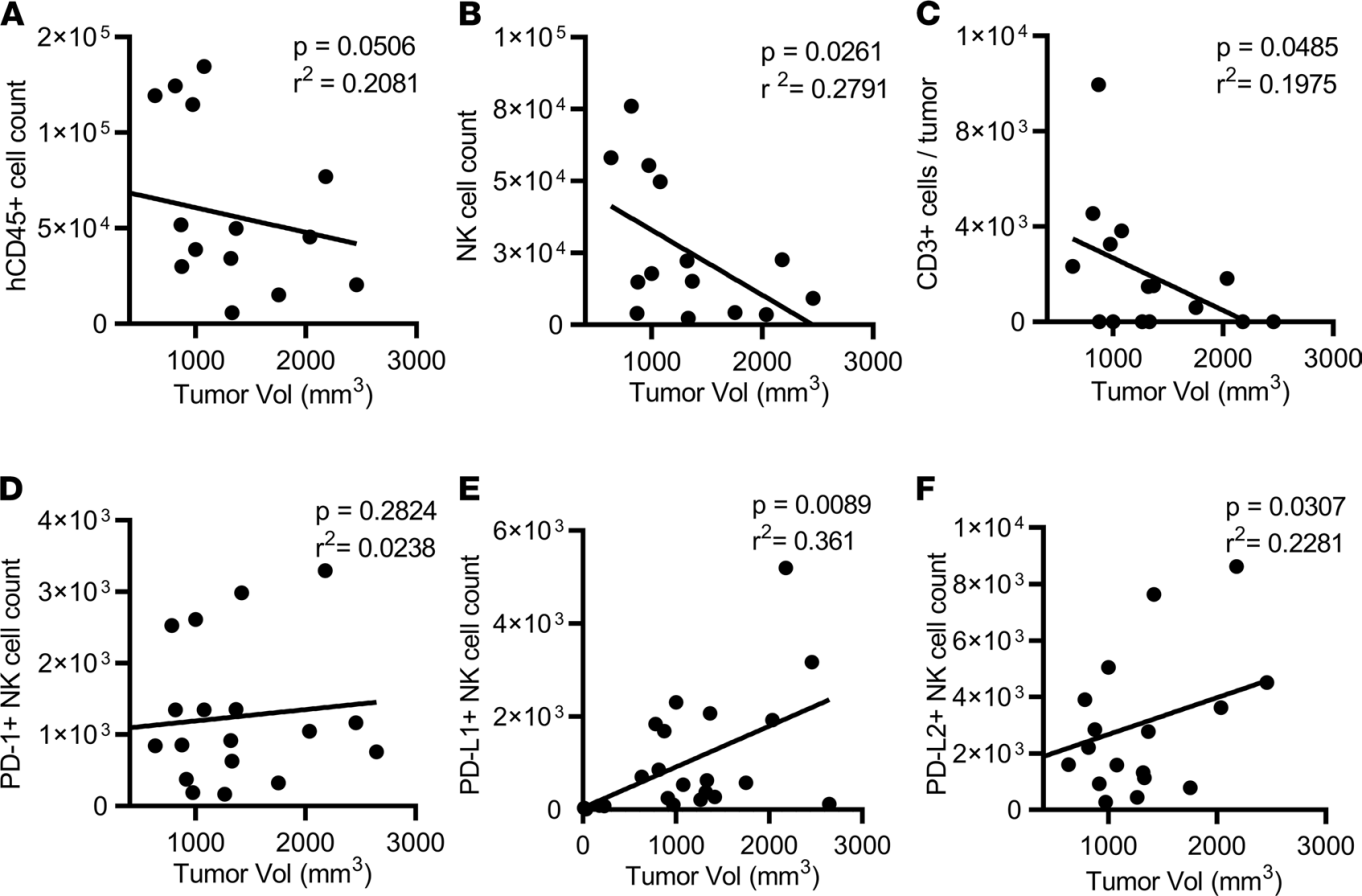
Correlation of numbers of tumor-resident human immune cells and tumor size in TIL-PDX-LUAD mice 6 months posttransplant. (**A**–**D**) Linear regression plots demonstrating correlations between numbers of human (**A**) hematopoietic cells (CD45^+^), (**B**) NK cells (CD45^+^CD3^–^CD56^+^), and (**C**) conventional T cells (CD45^+^CD3^+^CD56^–^) or (**D**–**F**) indicated immunomodulatory receptors expressed on NK cells and tumor volumes 6 months posttransplant. Four unrelated donor cohorts were analyzed. Depending on cohort size, 3 to 6 TIL-PDX-LUAD mice of each cohort were analyzed, resulting in 14 to 22 data points total per marker/cell type. Correlations were computed using Pearson’s correlation coefficients, and *P* values are indicated for each correlation.

To determine residual antitumor immune function in our TIL-PDX model, we next examined the correlation between tumor growth and the presence of effector cells and their expression of PD molecules (Figure 6, A–F). We found an inverse correlation between tumor size and the presence of human immune cells (*P* < 0.0507; Figure 6A) and a significant inverse correlation between tumor size and NK cell count (Figure 6B), as well as T cell count (Figure 6C). We also found a positive correlation between tumor size and NK cells expressing PD-L1 and PD-L2 (Figure 6, E and F). NK cells expressing PD-1 did not correlate significantly with tumor growth, however (Figure 6D). Thus, transplanted tumors with a higher number of infiltrating NK or T cells were associated with smaller tumor size, while NK cells expressing PD-L1 or PD-L2 were correlated with larger tumors. These findings support known evidence that the degree of immune cell infiltration is a marker of prognosis in a variety of tumors.

### IL-15 stimulation prevents tumor escape from ICI and leads to sustained tumor regression.

**Figure 7:**
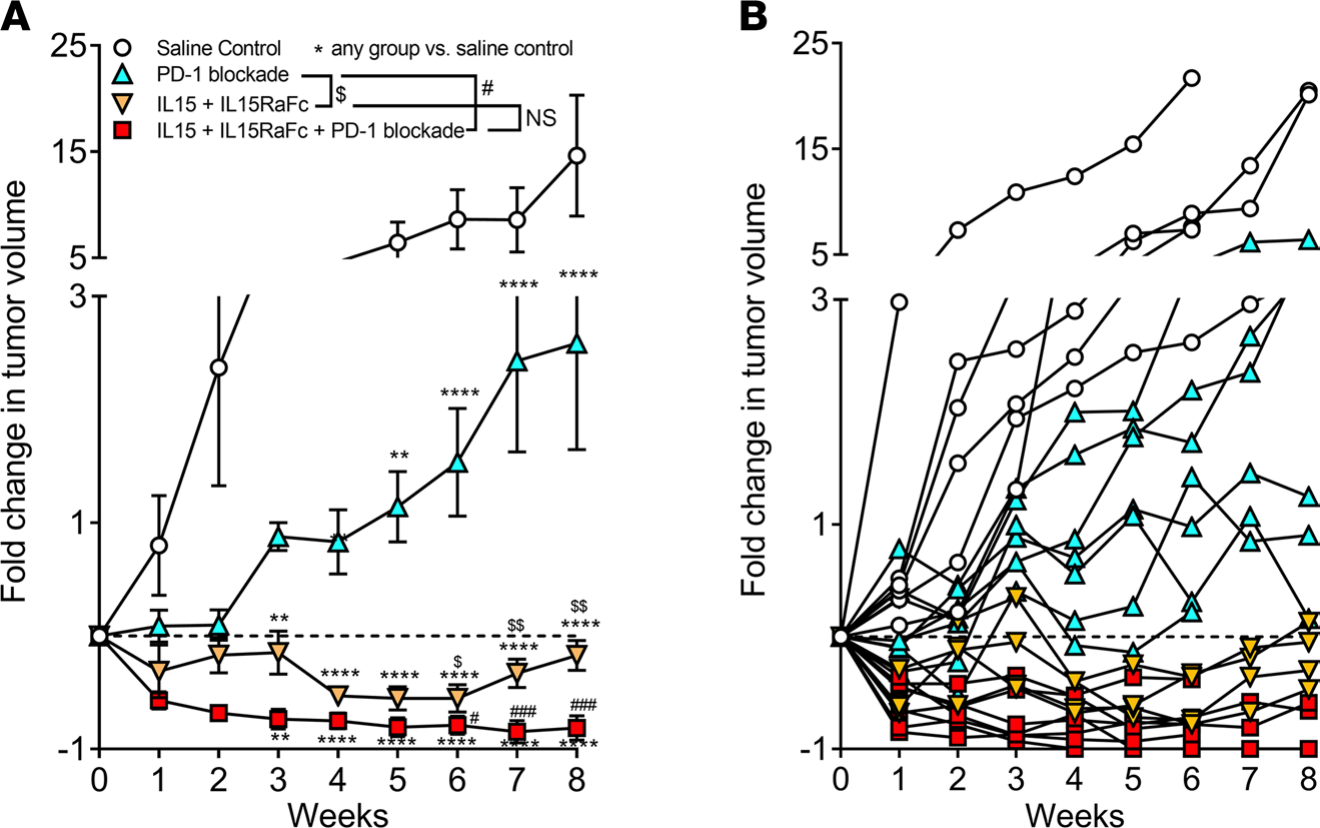
IL-15 stimulation prevents tumor escape from ICI, resulting in complete or partial remission. Fold change in tumor volume in TIL-PDX-LUAD mice treated with 100 μg/mouse blocking antibody against PD-1, 5 μg/mouse IL-15 + 2.5 μg/mouse IL-15RαFc, or both PD-1 and IL-15/IL-15RαFc as indicated by intraperitoneal injection at day 0 and every 7 days after that. Control mice received saline. Experiments were started when tumors were about 50 ± 14 mm^3^ with no statistical size difference between experimental and control groups at day 0 and tumor volumes measured weekly. (**A**) Mean fold change in tumor volume of each experimental or control group with SEM. (**B**) Mean fold change in tumor volume of TIL-PDX-LUAD mice with SEM. Three independent experiments were performed using 3 genetically unrelated tumor donor cohorts and data pooled for analysis. Depending on cohort size, 2 to 3 TIL-PDX-LUAD mice each were analyzed per group per cohort for *n* = 9 for saline control, *n* = 7 PD-1 blockade, *n* = 6 for IL-15 stimulation, *n* = 7 for IL-15 stimulation + PD-1 blockade. Data are represented as mean ± SEM; multiple *t* tests, *,^#^*P* < 0.05; **,^##^*P* < 0.005; ***,^###^*P* < 0.0005; ****P < 0.00005.

We hypothesized that immunotherapy aimed at restoring the activation of tumor-exhausted T and NK cells would elicit potent antitumor responses in one or both cytotoxic immune cell types. To achieve this goal, we chose a combination of PD-1 blockade and IL-15 stimulation as our combination immunotherapy approach, as both treatments were reported to be safe for humans (61). Also, IL-15 stimulation increases the expression of the activating receptors CD16 and NKG2D on NK cells and increases the activation, proliferation, cytotoxic activity, and survival of NK cells and CTLs (79). We found that the combination of PD-1 blockade and IL-15 signaling resulted in the profound regression of transplanted LUAD in about one-half of treated TIL-PDX-LUAD mice. The other half experienced a partial response (Figure 7). Notably, transpresented IL-15 alone, but not PD-1 blockade, significantly reduced tumor burden in all treated TIL-PDX-LUAD animals. PD-1 blockade alone transiently prevented tumor growth, but after 2 weeks of therapy, tumors grew at a similar rate to untreated control tumors (Figure 7). The addition of IL-15 to PD-1 blockade completely abrogated tumor escape from ICI, leading to profound, prolonged tumor regression. These findings support a crucial role for adjuvant IL-15 treatment to ICI to prevent tumor immune escape.

**Figure 8:**
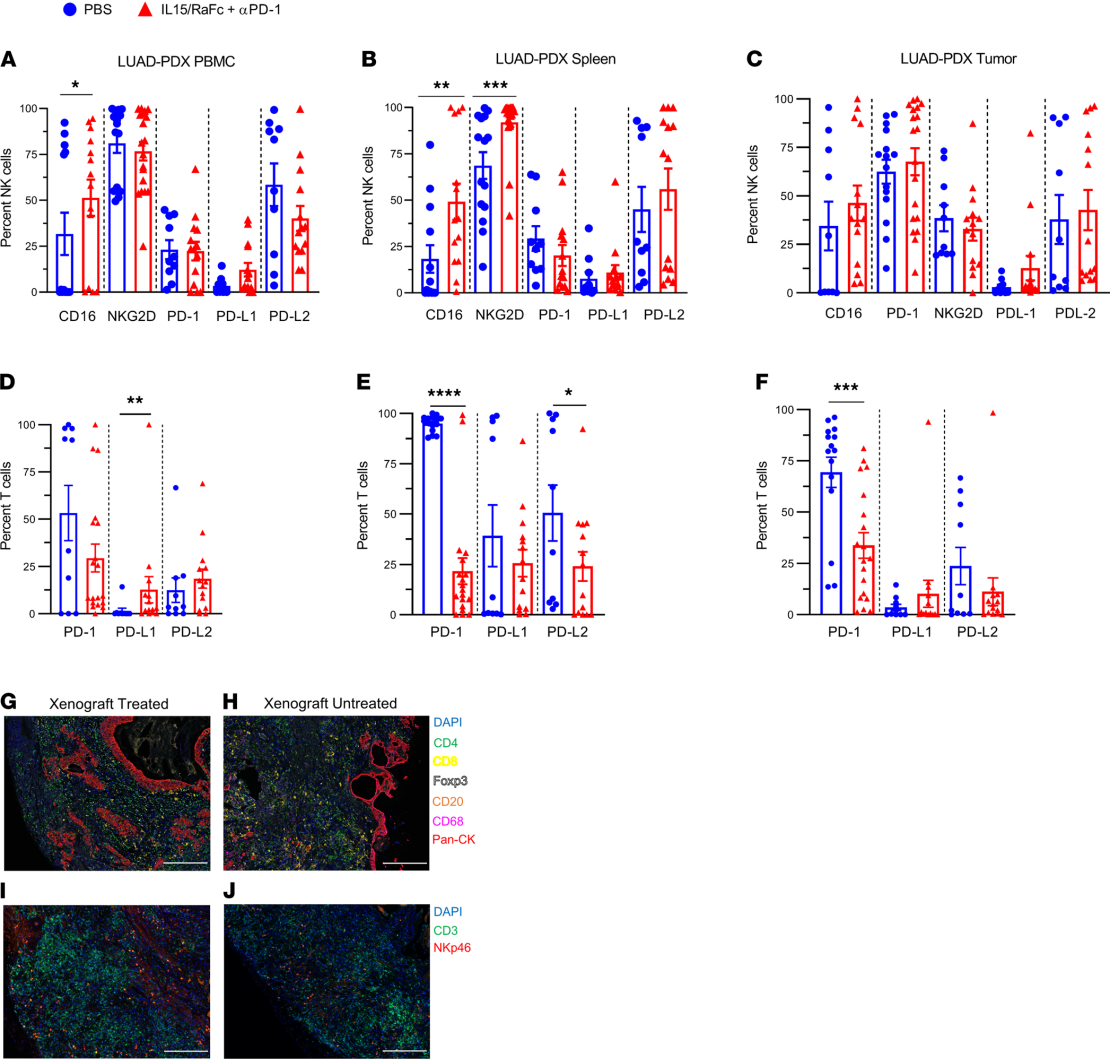
IL-15/RαFc and PD-1 blockade increase frequencies of effector NK cells and T cells. Frequencies of NK cells expressing CD16, NKG2D, PD-1, PD-L1, and PD-L2 as well as frequencies of T cells expressing PD-1, PD-L1, and PD-L2 status after 6 to 8 weeks of treatment with IL-15/RαFc + PD-1 blocking antibody or saline in TIL-PDX-LUAD tissues (**A** and **D**): PBMCs, (**B** and **E**) spleen, and (**C** and **F**) tumor. Seven-color (DAPI, CD4, CD8, Foxp3, CD20, CD68, pan-cytokeratin) IHC stain of TIL-PDX-LUAD tumor status after 3 weeks of (**G**) IL-15/RαFc + PD-1 blockade or (**H**) saline treatment. Three-color (DAPI, CD3, NKp46) IHC stain of TIL-PDX-LUAD tumor status after 3 weeks (**I**) IL-15/RαFc + PD-1 blockade or (**J**) saline. Scale bars: (**G**–**J**) 200 μm. Each data point represents 1 TIL-PDX-LUAD mouse tissue. Three genetically unrelated human donor cohorts were analyzed. Depending on cohort size, 4 to 7 TIL-PDX-LUAD mice each were analyzed for a total of 14 to 19 data points per tissue and cell type. Data are represented as mean ± SEM; paired *t* test between treatment and control. *P < 0.05; **P < 0.01; ***P < 0.001; ****P < 0.0001.

We then analyzed the effect of IL-15 and PD-1 blockade on the immune effector cell subsets using flow cytometry. Consistent with IL-15’s known mechanism of action, we observed a statistically significant increase in the proportion of CD16^+^ NK cells in PBMCs and spleens of TIL-PDX-LUAD–treated mice compared with control. NKG2D-expressing NK cells were also seen at higher proportions in TIL-PDX-LUAD mouse spleens (Figure 8, A and B), while their proportions remained unchanged in the tumor (Figure 8C). Furthermore, we observed a marked decrease of PD-1–expressing CD3^+^ T cells in the peripheral blood (Figure 8D), and CD3^+^ T cells showed a statistically significant decrease in the proportion of PD-1^+^ cells in response to IL-15 stimulation and PD-1 blockade in both the spleen and tumor of TIL-PDX-LUAD mice (Figure 8, E and F). Further supporting these findings, multiplex IHC revealed the continued presence of various immune subsets in treated and untreated xenografts, including CD4^+^ and CD8^+^ T cells and NK cells, among others (Figure 8, G–J). Taken together, these data demonstrate findings consistent with IL-15 stimulation and PD-1 blockade activating/disinhibiting NK cells and CTLs in our TIL-PDX-LUAD model.

### NK cells and CTLs are required for successful combination immunotherapy in the TIL-PDX-LUAD model.

**Figure 9:**
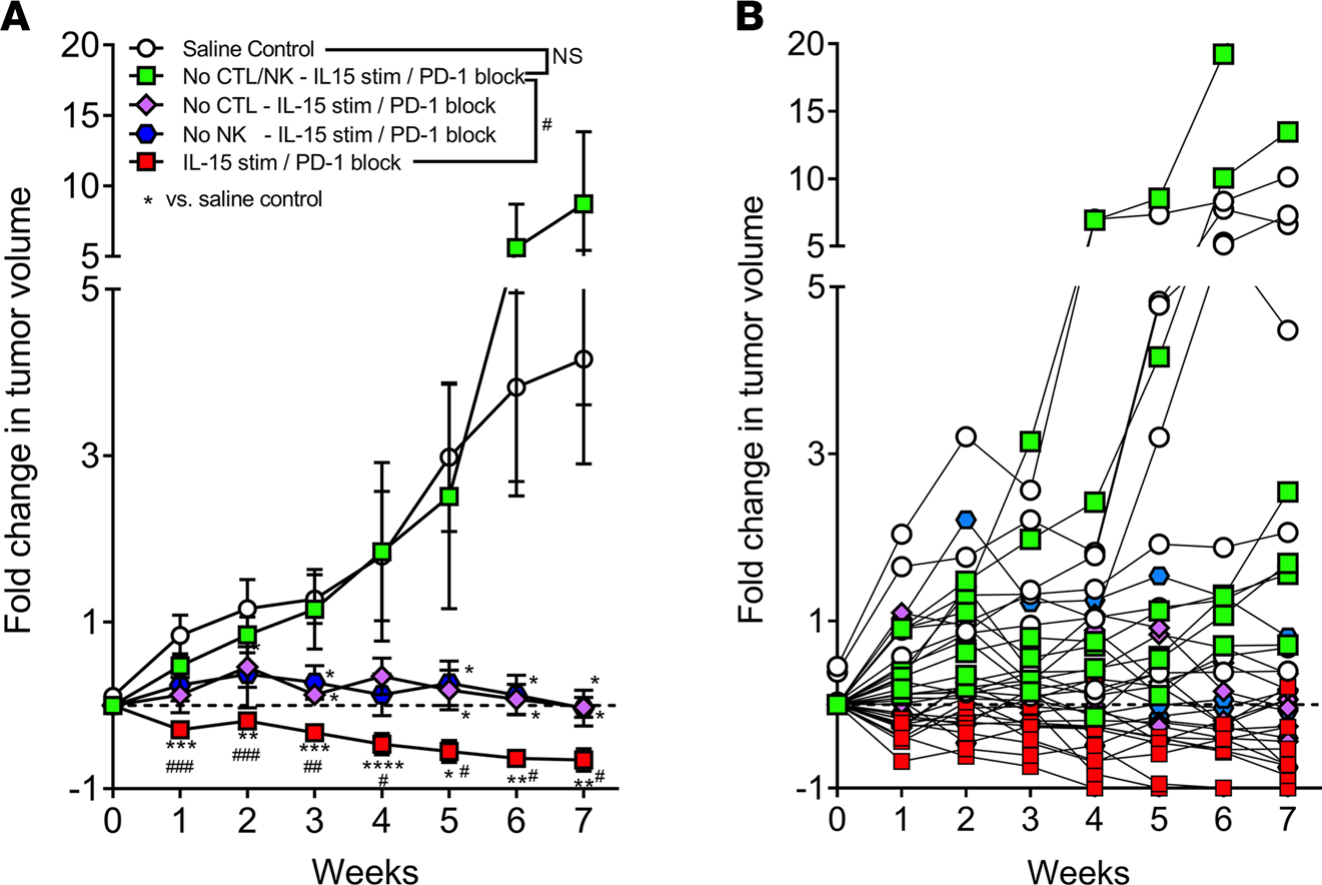
NK cells and CTLs are required for successful combination immunotherapy in the TIL-PDX-LUAD model. (**A**) Fold change in tumor volume in TIL-PDX-LUAD mice treated with 100 μg/mouse blocking antibody against PD-1 and 5 μg/mouse IL-15 + 2.5 μg/mouse IL-15RαFc by intraperitoneal injection at day 0 and every 7 days after that. Control mice received saline. Experiments were started when tumors were about 50 ± 14 mm^3^ with no statistical size difference between experimental and control groups at day 0 and tumor volumes measured weekly. Three days prior to the start of PD-1 blockade + IL-15/IL-15RαFc therapy (day –3) as well as on days 0, 7, 14, 21, 28, 35, 42, and 49, CTLs and/or NK cells were depleted using 100 μg/mouse of antibody specific to human CD8a (OKT-8) to deplete CTLs, and/or antibody specific to NKp46 (9E2) to deplete NK cells, as indicated. Control mice received saline. (**A**) Mean fold change in tumor volume of each experimental or control group with SEM. (**B**) Mean fold change in tumor volume of TIL-PDX-LUAD mouse with SEM. Three independent experiments were performed using 3 genetically unrelated tumor donor cohorts and data pooled for analysis. Depending on cohort size, 2 to 3 TIL-PDX-LUAD mice each were analyzed from each cohort for *n* = 8 for untreated control group, *n* = 9 for IL-15 stimulation + PD-1 blockade, *n* = 6 for IL-15 stimulation + PD-1 blockade and NK cell depletion, *n* = 6 for IL-15 stimulation + PD-1 blockade and CTL depletion. Data are represented as mean ± SEM; multiple *t* tests, ^#^,^$^*P* < 0.05; **,^$$^*P* < 0.005; ^###^*P* < 0.0005; ****P < 0.00005.

We hypothesized that CTLs and NK cells are key players behind the combined IL-15 and PD-1 blockade immunotherapy response in our TIL-PDX-LUAD model. To dissect their individual contributions to tumor regression, we depleted CTLs alone, NK cells alone, or both effector cell types using antibodies specific to CD8α (CTL depletion) or NKp46 (NK cell depletion). Depleted xenografts were then treated weekly with IL-15/IL-15RαFc + PD-1 blockade combination immunotherapy. We found that the depletion of either CTLs or NK cells resulted in stable disease. In contrast, depletion of both cell types unleashed rapid and lethal tumor growth, even in the presence of immunotherapy (Figure 9). Further strengthening these data, implantation of previously frozen tumors, whereby TILs were killed from the freezing, resulted in ineffective treatment with IL-15/IL-15RαFc + PD-1 blockade in tumor-bearing PDXs, as demonstrated by their tumors growing 5-fold (Supplemental Figure 8). We conclude that IL-15/IL-15RαFc + PD-1 blockade activates/disinhibits both CTLs and NK cells, that CTLs and NK cells are the main targets of this therapy, and that CTLs and NK cells are equally required for maximal antitumor response in human solid tumor as demonstrated in our TIL-PDX model of lung adenocarcinoma. We further conclude that our TIL-PDX-LUAD model not only enables much-needed efficacy evaluations of immunotherapies to reverse tumor-induced immune suppression effectively but additionally permits mechanistic studies to identify immune effector cells crucial to optimal immunotherapy in solid tumors.

## Discussion

Current PDX models lack a human tumor donor–matched, competent, functional, and mouse-tolerant immune system (73–77). One PDX model relies on serial transplantation of solid tumors into NSG mice to maintain genetic tumor diversity and allows for the evaluations of tumor genetics and chemotherapies (80). However, an infusion of donor-matched or mismatched PBMCs is required for immune system reconstitution, which results in the development of graft-versus-host disease (GvHD) within weeks of infusion (73). To circumvent GvHD, the second PDX model was generated by infusion of umbilical cord–derived hematopoietic stem cells (UB-HSCs) into immunocompromised mice before tumor engraftment, resulting in the development of mouse-tolerant T cells (73). However, while GvHD was avoided, UB-HSC–derived T cells were not restricted to human HLA because of the lack of human thymic education, resulting in artifactual CTL responses. Further, NK cells may or may not be inhibited by tumor-expressed HLA, depending on the nature of UB-HSC-NK cell killer cell immunoglobulin-like receptor expression, which would result in either artificial inhibition or augmentation of NK cell antitumor responses (73). These hurdles limit such PDX models’ usefulness to develop and test relevant immunotherapy against human solid tumors.

Here, we introduce a TIL-PDX model of human solid tumor with a TIL-derived, syngeneic, functional, and mouse-tolerant immune system. In our model, long-term immune reconstitution does not require PBMC or stem cell infusion, thus avoiding GvHD or the induction of allogeneic immune cells. Furthermore, our TIL-PDX model does not necessitate in vitro pre-expansion of human TILs or cytokine treatment of tumor-engrafted NSG mice. Instead, we achieve long-term immune reconstitution by implanting an undisrupted, treatment-naive piece of LUAD or PDAC. The accompanying TILs are then seen to repopulate the periphery of the recipient NSG mouse over time. About 89% of transplanted TIL-PDX-LUAD and about 83% of TIL-PDX-PDAC mice harbor tumors and reconstitute long term with tumor-associated effector and regulatory cells. In reconstituted PDX-LUAD and -PDAC mice, CTLs and NK cells replicated and conserved the exhausted phenotype of the original human tumor donors’ effector cells from which they were derived. These effector cells also persisted for many months after engraftment.

The NK cells in our model are mature NK cells derived from a tumor environment. They differ in phenotype from human PB NK cells (Figures 1 and 2) and persist without the addition of human IL-15, mediating residual antitumor activity (Figure 6), while remaining responsive to therapeutic IL-15 stimulation (Figures 7–9). Our data are in contrast to the observed lack of functional NK cell engraftment in HSC-derived humanized mouse models (81). In NSG mice, infused HSCs eventually develop into NK cells; however, human IL-15 is crucial for this development (82). The lack of IL-15 in HSC-derived humanized mouse models is the likely reason for the observed lack of mature NK cells (83).

By closely replicating an immune system derived from TILs of a human tumor in a mouse model, our model is best suited for preclinical studies of effectiveness and mechanisms of immunomodulatory therapies aimed at human tumors’ endogenous tumor microenvironment. We show proof of this principle here, whereby IL-15 stimulation significantly augmented ICI, as demonstrated by enhanced tumor regression (Figure 7). Interestingly, CTLs and NK cells are both targets of this combination immunotherapy and are sufficient and equally required for tumor regression. As such, our preclinical TIL-PDX model is a valuable resource for robust mechanistic and therapeutic studies in solid tumors. Furthermore, one may also study the tumor cells themselves in a system that likely replicates the original human environment as closely as possible and is amenable to further interrogation through genomic, metabolomic, or proteomic approaches.

We have observed that the expression of PD family members was tissue and cell specific. We found that most lung- and LUAD-resident NK cells from freshly resected human donor tissue expressed PD-1 as well as PD-L1 and/or PD-L2. However, the frequency of PD-1–, PD-L1–, and PD-L2–expressing NK cells is significantly higher in lung tumors compared with donor-matched nonmalignant lung tissues. In contrast, the same donor’s PBMC-derived NK cells had much lower proportions of PD-1–, PD-L1–, or PD-L2–expressing cells. Similarly, lung and LUAD-resident T cells expressed PD-1 and/or PD-L2, with an increased frequency observed in tumors compared with normal tissues. However, in contrast to NK cells, they did not express PD-L1. Furthermore, similar to our data on NK cells, many fewer PBMC-derived T cells expressed PD-1–, PD-L1–, or PD-L2–expressing cells. This is in line with several groups that have also reported PD ligand expression on NK cells and T cells (84–86). Further research is needed to clarify the significance of PD ligands’ expression on T cells, and NK cells, as much is not currently known.

An important advance of our TIL-PDX model is the long-term engraftment of a syngeneic, TIL-derived immune system that is functional and mouse tolerant and preserves CTLs’ and NK cells’ tumor-induced exhausted phenotype. Our TIL-PDX model also allows for the testing and mechanistic studies of immunotherapies in solid tumors. While several currently ongoing clinical trials are evaluating the efficacy of ICI monotherapies in localized and locally advanced solid tumor types, including NSCLC (NCT03425643; NCT02998528), ICI monotherapies are approved as a therapy for stage III NSCLC only after chemo-irradiation (87) and not as a primary treatment. In line with our long-term goal to use TIL-PDX mice to develop effective primary combination immunotherapies, we examined if the addition of IL-15 stimulation to PD-1 blockade augments the antitumor response of tumor-exhausted CTLs and NK cells in our TIL-PDX-LUAD model. To our surprise, IL-15 stimulation was far superior to PD-1 blockade, which only temporarily halted tumor growth before immune escape and resumption of growth at a rate similar to untreated tumors. In contrast, IL-15 stimulation resulted in tumor regression in all mice. Notably, the combination of IL-15 stimulation and PD-1 blockade resulted in the most pronounced tumor regression in all treated TIL-PDX-LUAD mice, demonstrating that IL-15 stimulation prevents tumor escape from ICI and augments antitumor immunity. These data may be crucial for treating solid tumors for which ICI is of limited benefit, such as neoantigen-poor tumors like PDAC. In PDAC, CTL exhaustion can be prevented or reversed by PD-1 and/or PD-L1 blockade, but this approach frequently leads to immune escape through the emergence of MHC-I–null pancreatic cancer cells evading CTL killing (88). However, MHC-I downregulation may be advantageously exploited through NK cell recognition and lysis of cells lacking MHC-I expression (“missing self”). IL-15 stimulation to ICI is likely safe, as demonstrated by a recent phase I clinical trial that established that IL-15 stimulation and PD-1 blockade are both safe and beneficial to a portion of NSCLC patients who had failed conventional therapies (61). However, albeit understandably, this study (61) was conducted with a small number of patients for which IL-15 stimulation and PD-1 blockade were not their primary therapy, and no mechanistic studies could be performed.

To understand how our combination immunotherapy induced such strong regression of LUAD in TIL-PDX mice, we explored the individual role of CTLs and NK cells in antitumor immunity upon IL-15 stimulation and PD-1 blockade through selective depletion experiments. Interestingly, we found that both cell types were equally required and that their antitumor immunity synergizes, leading to profound regression of human solid tumors when together. Our findings demonstrate the unexpected plasticity of exhausted NK cells and CTLs in the tumor microenvironment that can be harnessed and redirected therapeutically to combat tumors in vivo jointly. This is consistent with previous reports in mice, one of which identified NK cell and CTL cooperation as crucial for controlling solid tumors (8, 9). The other demonstrated the effective augmentation of NK cell– and CTL-mediated antitumor activities through combined PD-L1 blockade and IL-15 stimulation in a murine model of solid tumor (89). In agreement with these data obtained in mice, our data demonstrate that IL-15 stimulation, when administered with ICI, effectively activates human antitumor activity in NK cells and CTLs. It is worth mentioning that NK cell contribution to tumor eradication may be even more significant than what our data suggest, as NK cell depletion with the commercially available antibody against NKp46 targets only NKp46-domain 1–expressing NK cells, leaving NKp46-domain 2–expressing NK cells intact. As such, future studies on the role of specific NK cell subsets in the rejection of solid tumors in response to immunotherapy are needed to understand the precise roles human NK cell subsets play in solid tumor rejection.

A significant benefit of our TIL-PDX model is that it allows the study of the human immune response to genetically diverse human tumors from both male and female human donors, in contrast to mouse tumor models, in which a specific mutation or combination of mutations resembles a subset of human tumors (90). Therefore, it is noteworthy that our therapy generally worked well with any tumor donor and across individual TIL-PDX mice of a given cohort (7 cohorts total with *n* = 20 saline control and *n* = 18 IL-15 stimulation + PD-1 blockade TIL-PDX-LUAD mice), given the expected genetic tumor heterogeneity within a single tumor and the human cancer patient population. Our data suggest that this model may work well to study immunotherapy across a diverse human population.

Limitations to this model are inherent to the fact that the reconstituted human immune system in the NSG mouse is entirely derived from the implanted piece of human tumor. Therefore, immune reconstitution will naturally vary somewhat from one tumor specimen to another. Factors to consider are any prior therapies the patient may have undergone, specifically cytoreductive treatments such as chemotherapy and/or radiotherapy, as these would reduce the number of transplanted TILs.

In summary, our new preclinical model provides a valuable resource for a multitude of mechanistic and therapeutic studies not previously possible due to the lack of an appropriate translational model. Using solid tumor TIL-PDX mice, we provide strong preclinical evidence for the use of combination immunotherapy to treat solid tumors and highlight the importance of NK cell–CTL cooperation in therapies frequently thought to target T cells exclusively.

## Methods

### Study design.

The goals of this study were to develop a PDX model in which (a) tumor and immune cells were from the same human donor; (b) relevant human immune effector and suppressor cells persisted long term in PDX mice; (c) human immune cells retained their tumor-exhausted phenotype, allowing for tumor growth; and (d) human immune cells did not cause GvHD. Having developed such a model, this study’s next goal was to develop a safe and effective immunotherapy preventing solid tumor immune escape from ICI, leading to tumor regression. Having developed such a therapy, this study’s remaining goal was to determine whether NK cells and/or CTLs were required for immunotherapy-elicited tumor regression. The numbers of human donors and PDX mice and statistical data analyses are indicated in each corresponding data figure’s legend.

### Mice.

NOD/SCID/IL2Rγ_c_-KO (NSG) mice were purchased from The Jackson Laboratory. Mice were cared for in the animal facilities of either the Center for Comparative Medicine at Baylor College of Medicine (BCM), Houston, Texas, USA, or the animal facilities of The Scripps Research Institute (TSRI), La Jolla, California, USA. All protocols were approved by the Institutional Animal Care and Use Committees of the respective institutes, and experiments were performed according to the Animal Welfare Act guidelines.

### PBMCs from LUAD and PDAC donors.

Ficoll-Paque (GE Healthcare, now Cytiva) was used to isolate PBMCs from PB according to the manufacturer’s instructions. The lymphocyte layer was removed, washed, and counted, and immune cells were stained with the indicated antibodies and analyzed using multiparametric flow cytometry.

### Tumor transplantation.

Freshly dissected, small, undisrupted pieces of LUAD or PDAC were sectioned into areas of 2 to 4 mm^3^, which were transplanted subcutaneously into 6- to 8-week NSG mice that were sex-matched to the human tumor donor. A digital caliper was used to measure tumor sizes weekly after transplant. Tumor volumes were calculated using the following formula: ½(L × W^2^), where L is the length and W is the tumor’s width.

### Processing of tissues.

TIL-PDX mice were euthanized when their tumors exceeded 100 mm^3^ in volume or at the experimental time points as indicated in the Results section. Blood was collected from TIL-PDX mice either by submandibular bleed or by cardiac puncture after euthanasia, and immune cells were isolated using Ficoll-Paque gradient centrifugation. Tumors and spleens were excised from TIL-PDX mice, and single-cell suspensions were generated by mechanical disruption of tissues followed by filtering of cell suspensions through 40 μm nylon mesh. Immune cells were separated by density gradient centrifugation using Ficoll-Paque. Layers of immune cells were separated from gradients and washed before proceeding with antibody staining.

### Flow cytometry.

Immune cells were treated with human- and mouse-specific Fc Block and then stained using indicated combinations of fluorescent antibodies listed in Supplemental Table 1 and identified based on their combination of markers expressed (Supplemental Figure 9). For *FOXP3* staining, Transcription Factor Staining Buffer Kit (Tonbo Biosciences) was used according to the manufacturer’s protocol. Fluorescence minus one controls were generated using a mix of human and murine PBMCs to distinguish between positive and negative cell populations. Samples were analyzed on either an LSR Fortessa (BD Biosciences) or a 5-laser Aurora (Cytek) and raw data analyzed further using FlowJo (version 10.4.2).

### Histopathology staining.

Tumors excised from TIL-PDX mice were fixed in 10% formalin, before H&E staining for histopathological analysis was performed at fee for service by the Pathology Lab Services of TSRI, La Jolla, California, USA.

### Multiplex IHC staining.

IHC assays were performed on 5 μm thick sections using the Opal 7 Solid Tumor Immunology Kit (Akoya Biosciences), which contained antibodies against CD4, CD8, CD68, CD20, Foxp3, and pan-cytokeratin. The assay was performed according to the manufacturer’s protocol. Slides were then mounted with ProLong Diamond Antifade Mountant (Thermo Fisher Scientific) before acquisition using Vectra Polaris (Akoya Biosciences). Visualization was carried out using PerkinElmer’s Phenochart software and inForm advanced image analysis software. NKp46 antibody was purchased from R&D Systems, Bio-Techne, clone 195314, catalog MAB1850. CD3 antibody was purchased from Thermo Fisher Scientific, clone SP7, catalog RM-9107-S0.

### Immunotherapy of TIL-PDX mice.

Six to 8 weeks posttransplantation, TIL-PDX mice with tumors about 50 mm^3^ in size were injected intraperitoneally (i.p.) once a week every week with IL-15 Receptor alpha Fc Chimera (R&D Systems, Bio-Techne; 2.5 μg/mouse) and recombinant human IL-15 (BioLegend, 5 μg/mouse). For immune blockade of PD-1, TIL-PDX mice were injected i.p. weekly with 100 μg of anti–human PD-1 (BioXcell, J116). All treatments were administered together on the same day.

### NK cell and CTL depletion in vivo.

To deplete human NKp46-domain 1–containing NK cells and CTLs, TIL-PDX mice were injected i.p. with 100 μg of antibodies against specific human NK cells expressing NKp46-domain 1 (BioLegend, 9E2) and/or human CD8α (BioXcell, OKT-8) 3 days before the first treatment and concurrently with subsequent treatments.

### Statistics.

All statistical analyses were calculated using GraphPad Prism 7 (GraphPad Software). The unpaired, 1-tailed Welch’s *t* test was used to compare 2 groups of unpaired data. The paired, 1-tailed *t* test was used when comparing 2 groups of paired data. The 1-way ANOVA with post hoc Tukey’s test was used to compare 3 or more groups of data. The Pearson correlation coefficient was computed to identify correlations. Data are presented as mean ± SEM. Statistical significance was set at *P* *<* 0.05, and individual *P* values are stated in each figure’s legend.

### Study approval.

All human PB, adult LUAD, lung, pancreas, and PDAC tissues were obtained with written informed consent, and the protocols were approved for use by the NIH and by BCM’s and TSRI’s Institutional Review Boards for the Protection of Human Subjects, and in accordance with the Declaration of Helsinki. For all animal experiments, all protocols were approved by the Institutional Animal Care and Use Committees of the respective institutes, and experiments were performed in accordance with the Animal Welfare Act guidelines.

## Author contributions

Conceptualization was performed by SP. Formal analysis was performed by DTL, TRH, SAA, and SP. Funding acquisition was performed by SP, GVB, FK, and TRH. Investigation was performed by DTL, TRH, BB, GVB, SAA, CZ, RN, and SP. Methodology was designed by DTL, TRH, BB, GVB, RN, and SP. Project administration was performed by SP. Resources were provided by BB, GVB, and JJT. Supervision was performed by SP. Validation was performed by DTL, TRH, SAA, RN, and SP. Visualization was performed by SP. Writing was performed by SP, TRH, and FK. Statement of the method used in assigning the authorship order among co–first authors: DTL and TRH share the title of first authors. The original manuscript was submitted for peer review in 2019, with DTL as the sole first author. At that time, DTL was no longer a member of the Paust Laboratory due to moving from Baylor College of Medicine to TSRI in September 2018. The reviewers requested substantial revisions. Because these revisions could not be performed by DTL, TRH reestablished the LUAD-TIL-PDX at TSRI, performed the reviewer-requested experiments, and analyzed data, and SP and DTL revised the manuscript and addressed the reviewer comments. Thereby, DTL’s and TRH’s contributions have equally contributed to the acceptance of the revised manuscript for publication, and they will share the co–first author position. DTL’s name is listed first, as DTL contributed more data than TRH to the final version accepted for publication.

## Supplementary Material

Supplemental data

## Version 1 06/03/2021

In-Press Preview

## Version 2 07/08/2021

Electronic publication

## Version 3 07/22/2021

The images for Figures 7 and 9 were swapped. They are now corrected.

## Version 4 07/26/2021

The P values for Figures 7 and 9 were corrected.
