# Supplementary material for: Natural killer cells and cytotoxic T lymphocytes are required to clear solid tumor in a patient-derived xenograft
Source: JCI Insight. 2021 Jul 8;6(13):e140116. doi: 10.1172/jci.insight.140116 (PMC8410059; doi:10.1172/jci.insight.140116)
Supplement: Supplemental data [file jciinsight-6-140116-s120.pdf]

Supplemental Figure 1

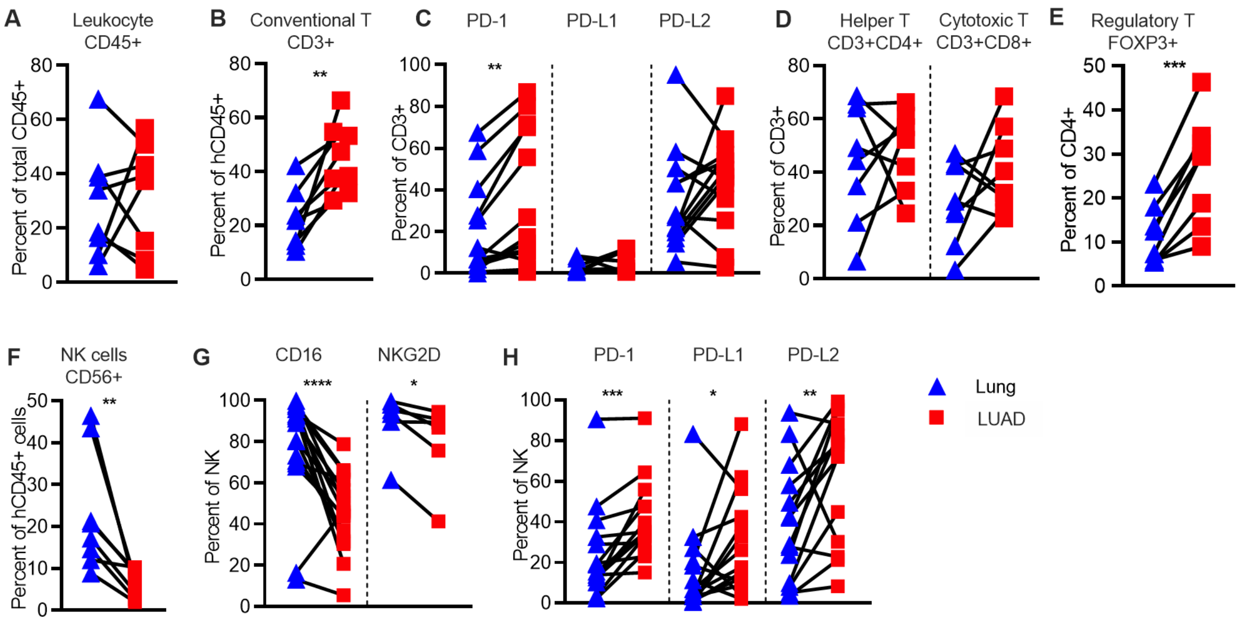

Supplemental Figure 1. Higher proportions of NK and T-cells express PD-molecules in LUAD compared to normal lung across genetically unrelated human donors. Flow cytometry staining of freshly excised human donor LUAD compared donor-matched normal lung tissue. (**A**) Percent of human CD45 cells, (B) percent of CD3 T-cells, as well as their expression of (C) PD-1, PD-L1, and PD-L2. (D) Frequencies of CD4 and CD8 T-cells as prevent of CD3 T-cells. (E) Frequencies of T-regs as a percent of CD4 cells. (F) Frequency of NK cells as a percent of human CD45 cells, their expression of (G) CD16, NKG2D, and (H) PD-1, PD-L1, PD-L2. Each paired data are from a genetically unrelated human donor. Paired t-test, *p < 0.05; **p < 0.01; ***p < 0.001.

Supplemental Figure 2

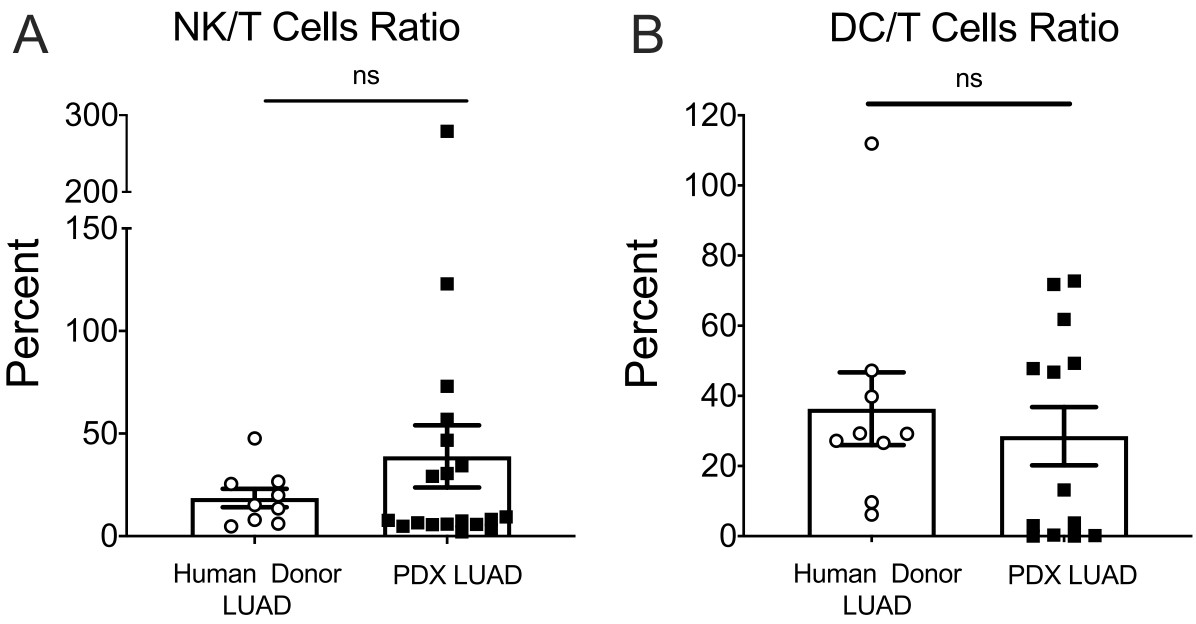

Supplemental Figure 2: Immune cell ratios are preserved in TIL-PDX-LUAD. The ratio of NK to T-cells **(A)** and DC to T-cells **(B)** in input human donor LUAD versus TIL-PDX-excised LUAD status-post implantation showing no statistically significant difference. Each data point represents a human donor (blue) or a TIL-PDX animal's tumor. Unpaired t-test; n.s. not significant ($p>0.05$).

Supplemental Figure 3

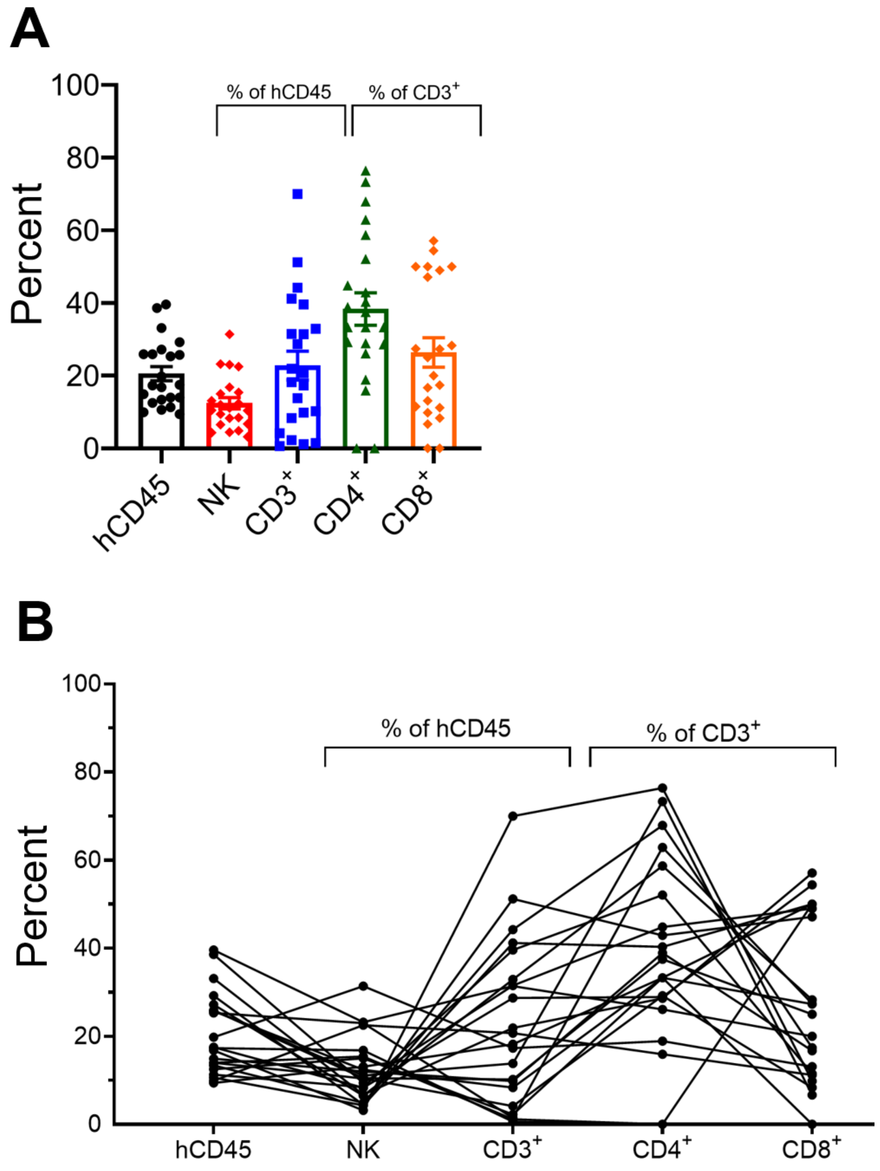

Supplemental Figure 3. Immune cell reconstitution across a cohort of PDX-LUAD mice 4-months post-implant. Flow cytometry of human immune cells from PBMC of a single TIL-PDX-LUAD cohort of 22 mice analyzed 4-months post-transplant. Frequency of human hematopoietic cells (human CD45$^+$ of total CD45$^+$), NK cells (CD45$^+$CD56$^+$CD3$^-$), conventional T cells (CD45$^+$CD56$^-$CD3$^+$), T helper cells (CD45$^+$CD56$^-$CD3$^+$CD4$^+$ CD8$^-$), and CTL (CD45$^+$CD56$^-$CD3$^+$CD8$^+$CD4$^-$). Each data point represents one TIL-PDX-LUAD mouse from a single human tumor-donor, N=22.

Supplemental Figure 4

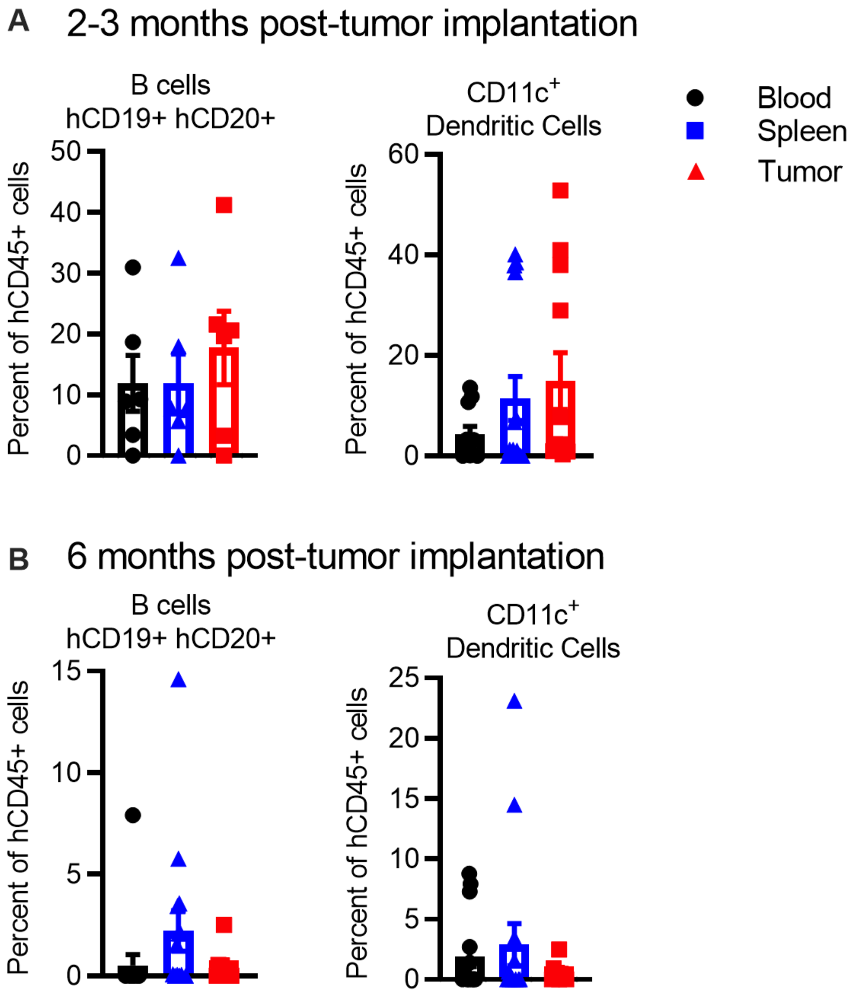

Supplemental Figure 4: Long-term reconstitution of TIL-PDX-LUAD mice with human B cells and dendritic cells (DC). Frequencies of Human B cells ($CD45^{+}CD3^{-}CD56^{-}CD19^{+}CD20^{+}$) and DC ($CD45^{+}CD3^{-}CD56^{-}CD19^{-}CD11c^{+}$) in PBMC, spleen, and excised tumor of TIL-PDX-LUAD mice (**A**) 2-3- and (**B**) 6-months post-tumor implantation, as determined by flow cytometry. Three genetically unrelated donor-cohorts were analyzed. Depending on cohort size, three to six TIL-PDX-LUAD mice of each cohort were analyzed at each time point, resulting in N= 11-15 data points per tissue.

Supplemental Figure 5

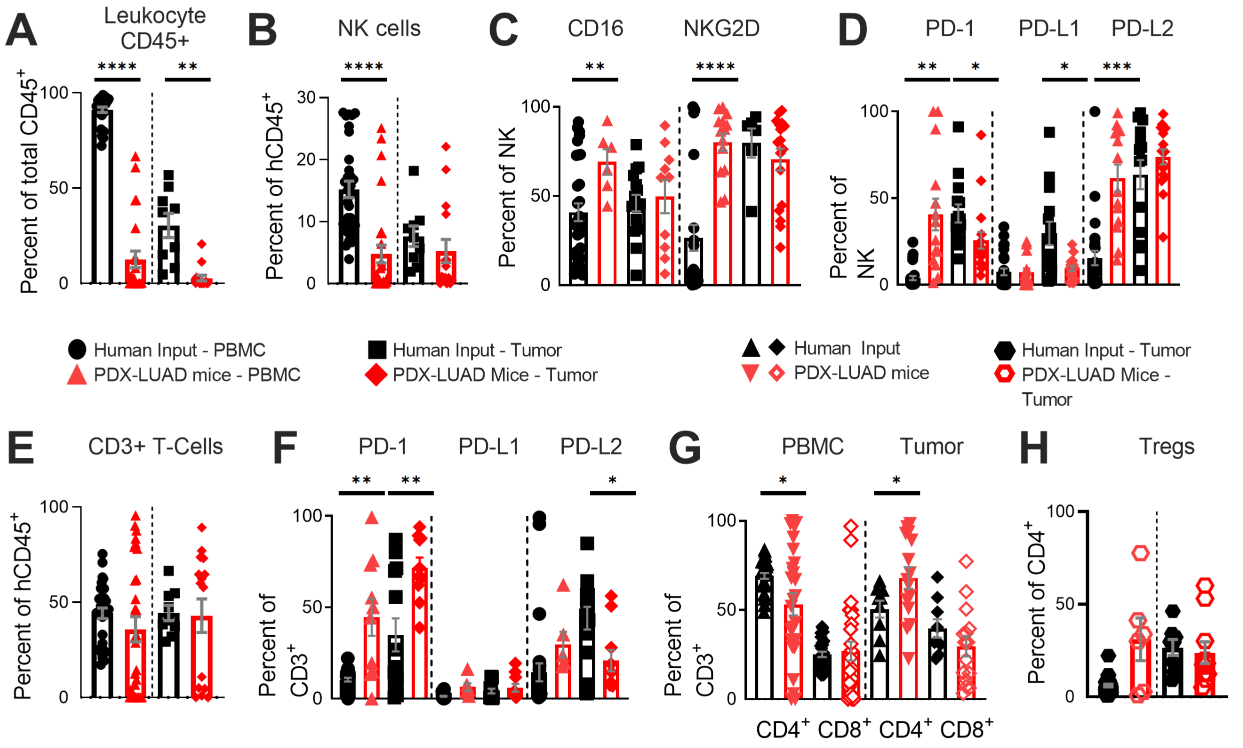

Supplemental Figure 5. Reconstituted TILs in TIL-PDX-LUAD retain human donor LUAD immune phenotypes. (**A-H**) Flow cytometry of human immune cells from LUAD tumor and PBMC of human donor compared to LUAD tumor and PBMC of implanted TIL-PDX-LUAD mice 2-months post-transplant. (**A**) Frequency of human hematopoietic cells ($CD45^+$), (**B**) NK cells ($CD45^+CD3^-CD56^+$), (**C**) expression of activating receptors CD16 and NKG2D, and (**D**) PD-1, PD-L1 and PD-L2 on NK cells. (**E**) Frequency of conventional T cells ($CD45^+CD3^+CD56^-$), and (**F**) expression of PD-1, PD-L1 and PD-L2 on conventional T cells. (**G**) Frequencies of T helper ($CD45^+CD3^+CD4^+CD8^-CD56^-$) and CTL ($CD45^+CD3^+CD4^-CD8^+CD56^-$), and (**H**) T-reg ($CD45^+$ $CD3^+CD4^+$Foxp-$3^+CD8^-CD56^-$) subsets. Human input PBMC or Tumor: Each data point represents one genetically unrelated human donor. TIL-PDX-LUAD mice: Five genetically unrelated donor-cohorts were analyzed. Depending on cohort size, three to six TIL-PDX mice of each cohort were analyzed at each time point, resulting in 15-30 data points total per cell type/marker and time point. Data are represented as mean SEM; one-way ANOVA with post-hoc Tukey test $*p < 0.05$; $**p < 0.01$; $***p < 0.001$.

Supplemental Figure 6

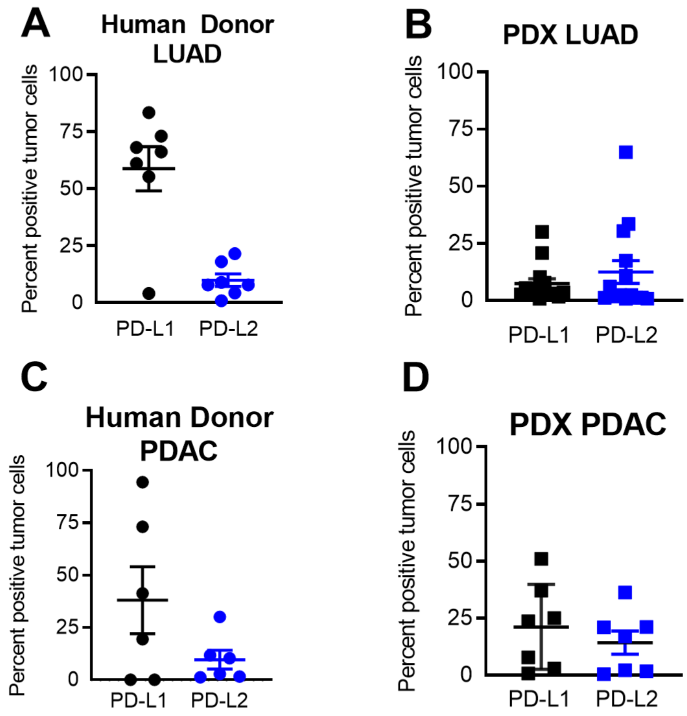

Supplemental Figure 6: PD-L1 and PD-L2-expression in freshly resected human donor LUAD and PDAC compared to TIL-PDX-LUAD and TIL-PDX-PDAC. Frequency of PD-L1 and PD-L2 expressing non-hematopoietic cells ($CD45^{-}$) in freshly dissected human tumors or TIL-PDX-LUAD or TIL-PDX-PDAC tumors four months post-transplant as determined by flow cytometry. N = 7 (LUAD) and N = 6 (PDAC) freshly excised tumor tissues were obtained from genetically unrelated human donors and analyzed by flow cytometry. Three genetically unrelated tumor donor cohorts were analyzed. Depending on cohort size, two to four TIL-PDX mice were analyzed per cohort for N = 7 PDX-PDAC and N = 10 TIL-PDX-LUAD mice. Unpaired t-test, p=0.0001 for PD-L1 freshly excised LUAD vs. TIL-PDX LUAD and not significant for all other comparisons. Freshly excised and TIL-PDX derived tumor tissues were not donor matched.

Supplemental Figure 7

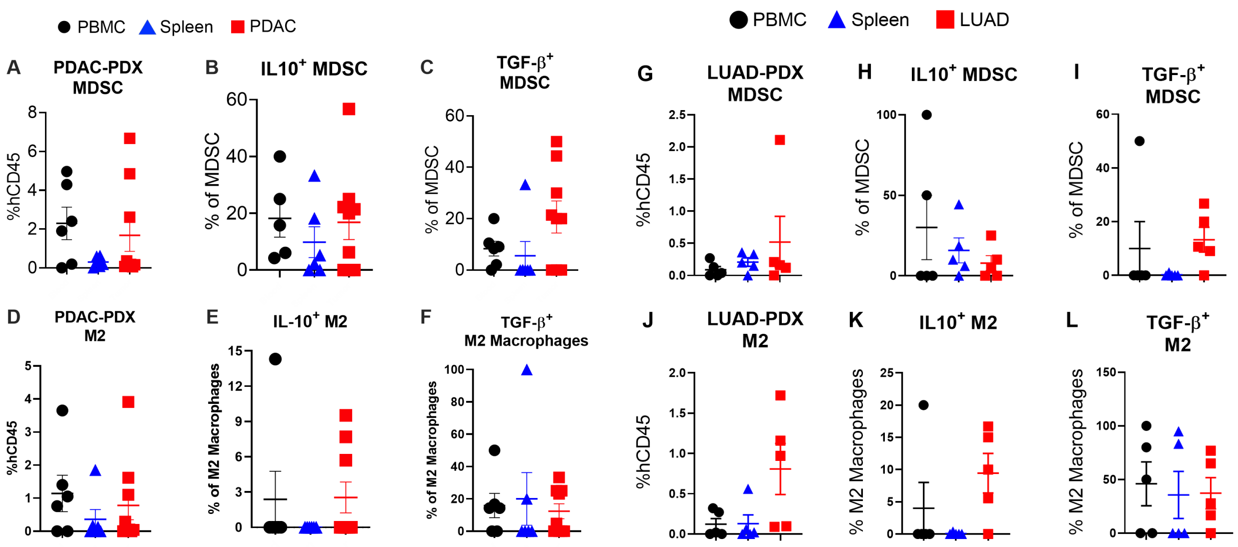

Supplemental Figure 7. Immuno-suppressive MDSC and M2 persistence in TIL-PDX-PDAC and TIL-PDX-LUAD mice. Frequency of (**A&G**) MDSC (human $CD45^+CD3^-CD56^-CD16^-$ $HLADR^{low/-}CD11b^+CD33^+$), (**B&H**) IL-10 producing MDSC and (**C&I**) TGF-beta-producing MDSC, as well as (**D&J**) M2 macrophages ($CD45^+CD3^-CD56^-CD14^+CD163^+$), (**E&K**) IL-10 producing M2 macrophages and (**F&L**) TGF-beta-producing M2 macrophages in PBMC, spleens and tumors of TIL-PDX-PDAC and TIL-PDX-LUAD mice 4-months post-transplant as determined by flow cytometry. Three (PADC) or two (LUAD) genetically unrelated tumor donor cohorts were analyzed. Depending on cohort size, two to three TIL-PDX-PDAC or mice were analyzed per cohort for N = 6-7 PDX-PDAC and N = 5 TIL-PDX-LUAD mice .

Supplemental Figure 8

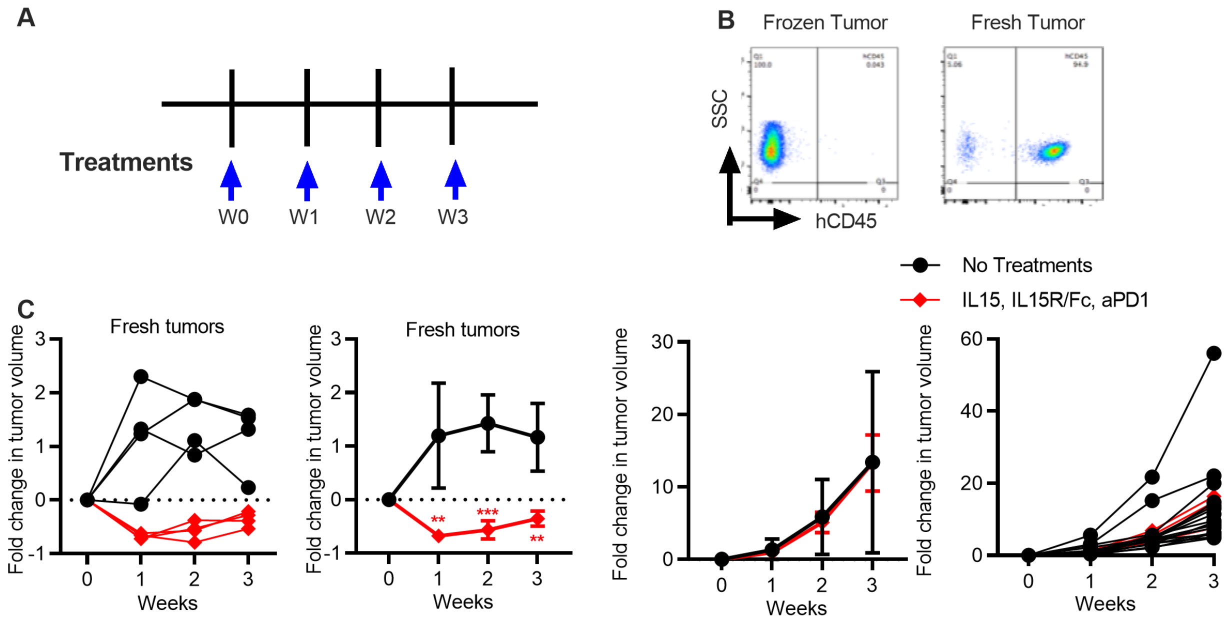

Supplemental Figure 8. Human immune cells are required for successful PD-1 blockade + IL-15/ IL15RaFc therapy. (**A**) Freshly harvested LUAD tumor tissue was transplanted into NSG mice (**B**), while the other half was frozen at -80°C for two weeks before being transplanted into NSG mice. Flow cytometry of TIL-PDX-LUAD mice demonstrating (**A**) the presence of human immune cells (human CD45^{+}) in recipients of fresh tumor tissue, compared to (**B**) the absence of human immune cells in recipients of frozen tumor tissue. TIL-PDX-LUAD mice were treated with either human IL-15 + human IL-15Ra Fc + PD-1 blocking antibody or left untreated, and tumor volumes were recorded weekly. N = 4 – 16 TIL-PDX-LUAD mice per group from two genetically unrelated donor cohorts, thus N= 8-32 mice total per group. Data are represented as mean SEM; Multiple t-test, *p < 0.05; **p < 0.01; ***p < 0.001.

Supplemental Figure 9

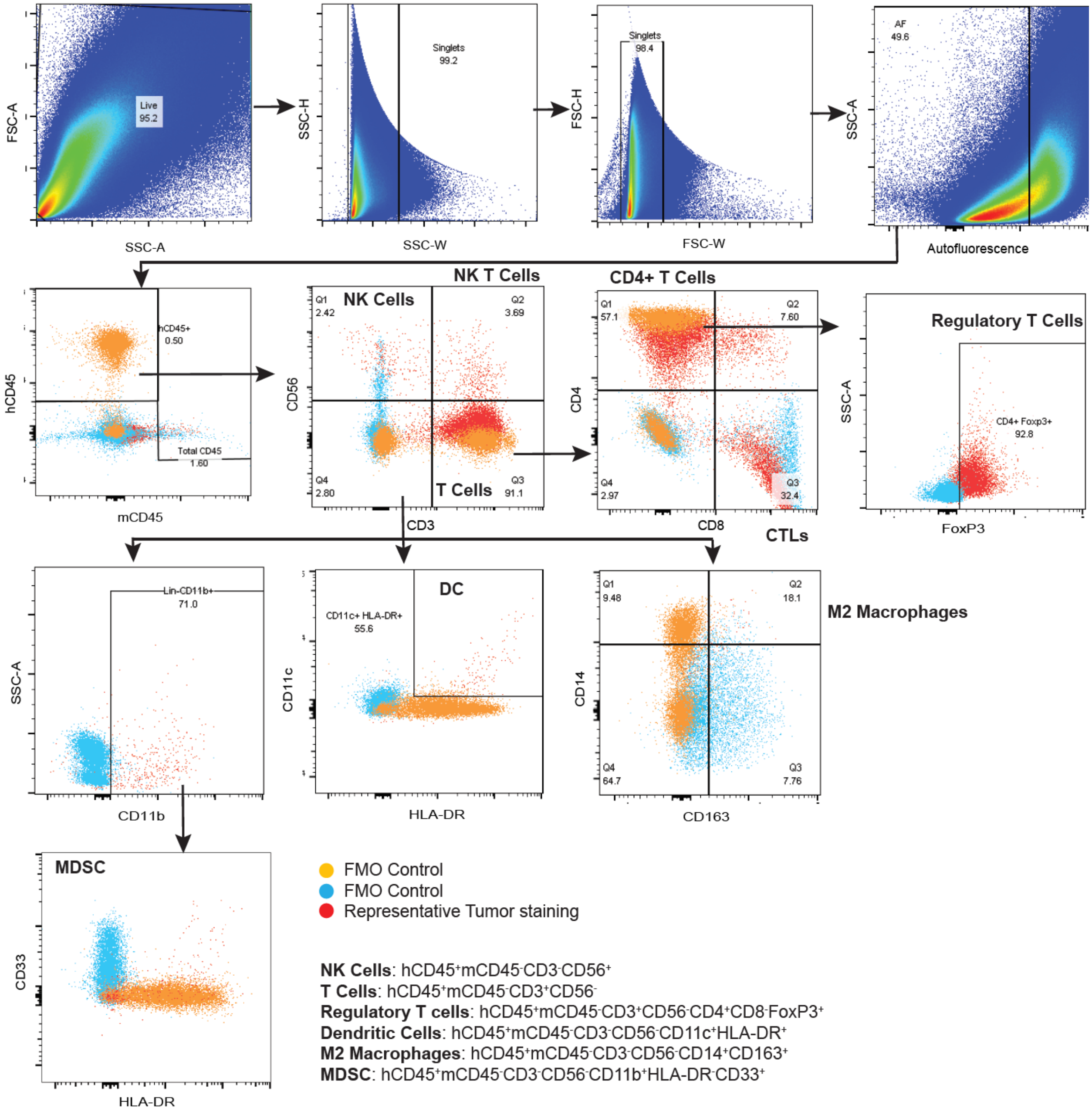

Supplemental Figure 9. Gating Strategy. Fluorescence minus one (FMO) controls for each fluorophore in each respective panel displayed in orange and teal. Representative TIL-PDX-LUAD staining in red. Markers of different cell types as detailed in the figure.

Supplemental Table 1

Antibody specificity	Fluorophore	Clone	Source	Identifier
**CD45**	Brilliant Violet 605™	HI30	Biolegend	304042
**CD3**	Brilliant Violet 711™	OKT3	Biolegend	317328
**CD3**	APC/Fire 750	UCHT1	Biolegend	300470
**CD56**	Brilliant Violet 421™	HCD56	Biolegend	318328
**CD56**	Brilliant Violet 570™	HCD56	Biolegend	318330
**CD4**	Brilliant Violet 650™	OKT4	Biolegend	317436
**CD4**	Brilliant Violet 750™	SK3	Biolegend	344644
**CD8a**	Brilliant Violet 785™	RPA-T8	Biolegend	301046
**CD11c**	PE/Cy7	3.9	Biolegend	301608
**CD11b**	PE/Dazzle™	ICRF44	Biolegend	301348
**CD16**	Brilliant Violet 510™	3G8	Biolegend	302048
**CD16**	PE/Cy7	3G8	Biolegend	302010
**CD19**	Alexa Fluor® 700	HIB19	Biolegend	302226
**CD20**	APC/Cy7	2H7	Biolegend	302314
**CD33**	FITC	HIM3-4	Biolegend	303304
**CD33**	BUV737	HIM3-4	BD	749230
**CD94**	PerCP Cy5.5	DX22	Biolegend	305514
**CD163**	Brilliant Violet 711™	GHI/61	Biolegend	333630
**PD-1**	Brilliant Violet 785™	EH12.2H7	Biolegend	329930
**PD-1**	Brilliant Violet 480™	EH12.1	BD Biosciences	566112
**PD-L2**	Alexa Fluor® 488	176611	R & D	FAB1224G
**PD-L2**	APC	MIH18	Biolegend	345508
**PD-L1**	Alexa Fluor® 700	130021	R & D	FAB1561N
**NKG2D**	Brilliant Violet 510™	1D11	Biolegend	320816
**NKG2D**	Super Bright 436	1D11	ThermoFisher	62-5878-42
**FOXP3**	Alexa Fluor® 647	150D	Biolegend	320014
**FOXP3**	Alexa Fluor® 532	PCH101	ThermoFisher	58-4776-42
**HLA-DR**	Brilliant Violet 650™	L243	Biolegend	307650
**IL-10**	Alexa Fluor® 647	JES3-9D7	Biolegend	501412
**TGF-beta**	PerCP/eFluor 710	FNLAP	Thermofisher	46-9829-42
**CD45**	PE/Cy5	30-F11	Biolegend	103110
**CD45***	PE	30-F11	Biolegend	103106
**CD45***	PerCP/Cy5.5	30-F11	Biolegend	103132
**Fc Block ™**	N/A	N/A	BD Biosciences	564220
**Mouse Fc Block ™***	N/A	2.4G2	BD Biosciences	553142

*** murine specific antibodies, all others are specific to human antigen**
